# Single-Cell RNA-Seq Reveals Lineage and X Chromosome Dynamics in Human Preimplantation Embryos

**DOI:** 10.1016/j.cell.2016.03.023

**Published:** 2016-05-05

**Authors:** Sophie Petropoulos, Daniel Edsgärd, Björn Reinius, Qiaolin Deng, Sarita Pauliina Panula, Simone Codeluppi, Alvaro Plaza Reyes, Sten Linnarsson, Rickard Sandberg, Fredrik Lanner

**Affiliations:** 1 Department of Clinical Science, Intervention and Technology, Karolinska Institutet, and Division of Obstetrics and Gynecology, Karolinska Universitetssjukhuset, 141 86 Stockholm, Sweden; 2 Ludwig Institute for Cancer Research, Box 240, 171 77 Stockholm, Sweden; 3 Department of Cell and Molecular Biology, Karolinska Institutet, 171 77 Stockholm, Sweden; 4 Department of Physiology and Pharmacology, Karolinska Institutet, 171 77 Stockholm, Sweden; 5 Department of Medical Biochemistry and Biophysics, Karolinska Institutet, 171 77 Stockholm, Sweden

## Abstract

Mouse studies have been instrumental in forming our current understanding of early cell-lineage decisions; however, similar insights into the early human development are severely limited. Here, we present a comprehensive transcriptional map of human embryo development, including the sequenced transcriptomes of 1,529 individual cells from 88 human preimplantation embryos. These data show that cells undergo an intermediate state of co-expression of lineage-specific genes, followed by a concurrent establishment of the trophectoderm, epiblast, and primitive endoderm lineages, which coincide with blastocyst formation. Female cells of all three lineages achieve dosage compensation of X chromosome RNA levels prior to implantation. However, in contrast to the mouse, *XIST* is transcribed from both alleles throughout the progression of this expression dampening, and X chromosome genes maintain biallelic expression while dosage compensation proceeds. We envision broad utility of this transcriptional atlas in future studies on human development as well as in stem cell research.

## Highlights

• Transcriptomes of 1,529 individual cells from 88 human preimplantation embryos
• Lineage segregation of trophectoderm, primitive endoderm, and pluripotent epiblast
• X chromosome dosage compensation in the human blastocyst

## Graphical Abstract

**Figure:**
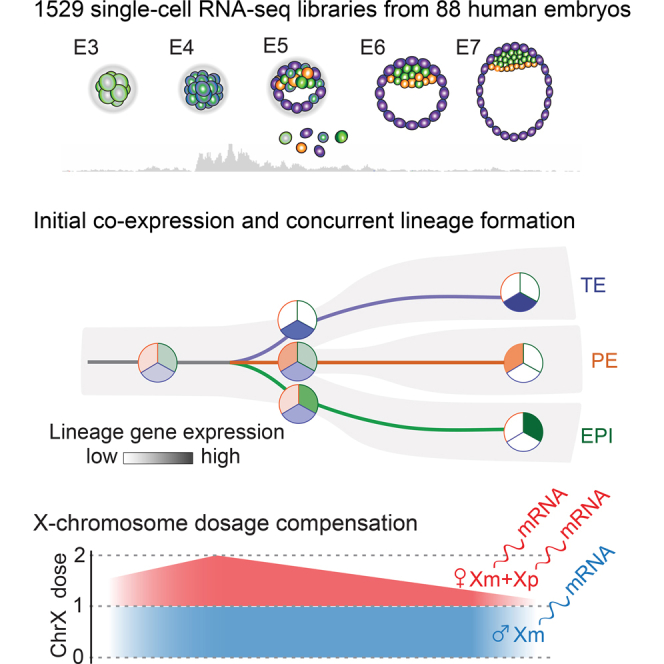

## Teaser

A comprehensive transcriptional map of human preimplantation development reveals a concurrent establishment of trophectoderm, epiblast, and primitive endoderm lineages and unique features of X chromosome dosage compensation in human.

## Introduction

During the first 7 days of human development, the zygote undergoes cellular division and establishes the first three distinct cell types of the mature blastocyst: trophectoderm (TE), primitive endoderm (PE), and epiblast (EPI) (Cockburn and Rossant, 2010). Although the molecular control underlying the formation of these lineages has been extensively explored in animal models, our knowledge of this process in the human embryo is rudimentary. In recent years, a limited number of studies have focused on translating conclusions from animal model systems to the human, providing many insights, but also revealing crucial species differences in the transcriptional and spatio-temporal regulation of lineage markers (van den Berg et al., 2011, Blakeley et al., 2015, Kunath et al., 2014, Niakan and Eggan, 2013), cell signaling responses (Kuijk et al., 2012, Roode et al., 2012, Yamanaka et al., 2010), as well as X chromosome inactivation (XCI) (Okamoto et al., 2011), thereby highlighting the need for studies of the human embryo.

In mouse, the TE and the inner cell mass (ICM) segregate first, and this is controlled by the opposing transcription factors caudal type homeobox 2 (CDX2) and POU domain class 5 transcription factor 1 (POU5F1, also known as OCTCT3/4) (Niwa et al., 2005). *Cdx2* is expressed ubiquitously at the 8-cell stage and then restricted to the outer cells of the 16-cell morula and the early 32-cell blastocyst. CDX2 repress POU5F1 expression in these outer cells, driving specification and maturation of the TE and ICM (Niwa et al., 2005). In the human, however, CDX2 protein is not expressed in the outer cells of the morula, but is only detected in the established blastocyst and coincides with POU5F1 in TE cells; thereby raising questions on the degree of conservation between the mouse and human TE-ICM maturation control mechanisms (van den Berg et al., 2011, Niakan and Eggan, 2013). Comparative studies on mouse, cattle, and human further suggest that the regulatory elements of *Pou5f1* diverged during mammalian evolution (van den Berg et al., 2011).

Further, it remains unclear when and how the divergence of the ICM into pluripotent EPI and PE occurs in human. Studies using antibody staining for lineage markers, such as NANOG, GATA4/6, and SOX17, encircled a rather wide range for this split; either coinciding with the blastocyst formation at embryonic day 5 (E5), or occurring during the late blastocyst stage at E7, just prior to implantation (Kuijk et al., 2012, Niakan and Eggan, 2013, Roode et al., 2012).

Another elusive facet of early human development is X chromosome dosage compensation. Eutherian mammals achieve X gene dose balance between females (XX) and males (XY) by transcriptional silencing of one X chromosome in female cells (Lyon, 1961). Failure to accomplish dosage compensation results in embryonic lethality (Goto and Takagi, 1998, Goto and Takagi, 2000). In mouse, imprinted inactivation of the paternal X chromosome initiates around the 4-cell stage (Deng et al., 2014a, Heard et al., 2004) and is mediated by *cis* coating of the silenced X chromosome with the long non-coding RNA (lncRNA) *Xist* (Clemson et al., 1998). The paternal X chromosome is thereafter kept inactivated in the TE and PE lineages, while reactivation and a round of random XCI takes place in the pre- and peri-implantation stage epiblast (Heard et al., 2004, Monk and Harper, 1979, Okamoto et al., 2004, Takagi and Sasaki, 1975). In contrast to the mouse, XCI is not imprinted in the human placenta (Moreira de Mello et al., 2010), which is a TE-derived tissue. Furthermore, the prevailing view is that human XCI does not take place until after implantation, or at least beyond the late blastocyst stage (Deng et al., 2014b), since RNA-FISH on X-linked genes, including *XIST*, show biallelic expression in most female TE and ICM blastomeres, even as late as E7 (Okamoto et al., 2011). Still, many aspects of the preimplantation regulation of the human X chromosome remain unexplored, as the available data rely mainly on allelic analyses of a few individual genes and direct assessments of female and male expression levels were previously not feasible.

Using single-cell RNA sequencing (RNA-seq) technology, we now provide a comprehensive resource, characterizing the transcriptional dynamics of progressive lineage specification and reveal X chromosome dosage compensation in the human preimplantation embryo.

## Results

### Single-Cell RNA-Seq Transcriptome Profiling of Human Preimplantation Embryos

**Figure 1:**
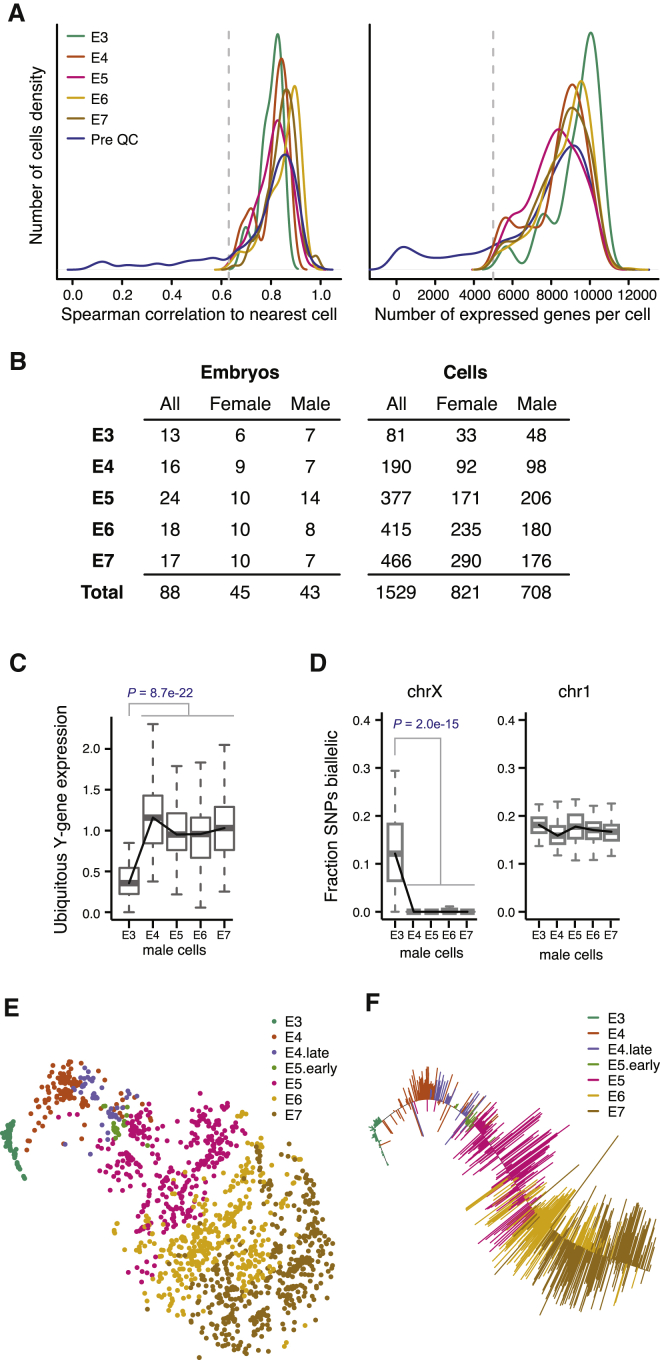
Single-Cell RNA-Seq Transcriptome Profiling of Human Preimplantation Embryos (A) Left: quality of single-cell RNA-seq experiments assessed as nearest-neighbor similarities between cells (maximum Spearman correlation per cell, using all cell-pairs and all genes). Right: histogram of the number of expressed genes per cell. Genes with RPKM ≥1 were considered expressed. The histograms were smoothed using a Gaussian kernel. (B) Number of embryos and cells per embryonic day (E3–E7) retained after quality filtering. (C) Expression-level boxplots for ubiquitously expressed Y chromosome genes in male cells, normalized to the median in stage E4–E7. p value, two-sided MWW. (D) Boxplots showing the fraction transcribed SNPs detected as biallelically expressed in male cells, shown for chromosome X and 1. p value, two-sided MWW. (E) Two-dimensional t-SNE representation of 1,529 single-cell preimplantation transcriptomes using the 500 most variable genes across all cells (according to Figures S2A and S2B). E3–E7 indicate the embryonic day and E4.late and E5.early indicate cells picked 4–6 hr later and earlier, respectively, than the other cells from that embryonic day. (F) A pseudo-time was assigned to each cell by fitting a principal curve to the cells in the two-dimensional t-SNE subspace (Figure 1E). ICM cells were excluded from the fit to let the principal curve better reflect time and minimize lineage-effects (Supplemental Experimental Procedures). See also Figures S1 and S2.

**Figure S1:**
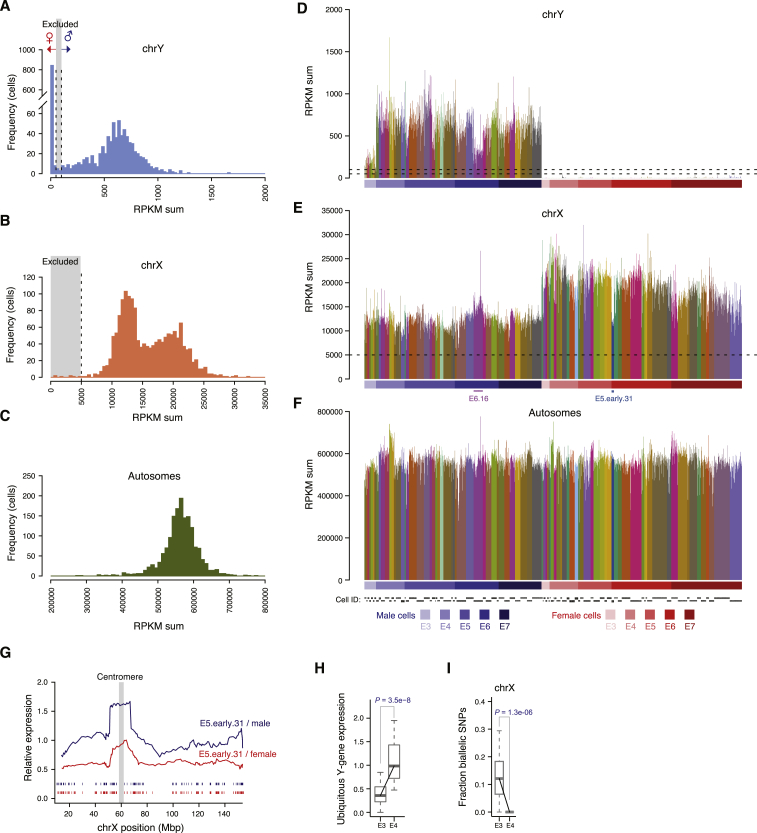
Sex Determination of Human Preimplantation Embryos, Related to Figure 1 (A) Histogram showing Y chromosome RPKM sum per cell on the x axis and cell frequency on the y axis. Based on the modality of this distribution, we classified cells with a Y chromosome RPKM sum below 50 as female, and above 100 as male (Supplemental Experimental Procedures). (B) Histogram of X chromosome RPKM sum per cell. (C) Histogram of autosomal RPKM sum per cell. (D–F) Barplots of chromosomal RPKM sums for sex-classified cells. Color indicates embryo. The expression from all genes located on each respective chromosome was used. (G) Moving expression average using a 25-nearest-genes window along the X chromosome for a female embryo with suspected X0 karyotype (E5.early.31) relative to the female E5 (red line) or male E5 (blue line) expression of other embryos. Based on its suspected X0 karyotype embryo E5.early.31 was excluded from all further dosage compensation analyses. (H) Expression-level boxplots for ubiquitously expressed Y chromosome genes per cell in cryo-preserved male E3 and cryo-preserved male E4 embryos, normalized to the median in stage E4-E7 (as in main Figure 1C). p value: two-sided Wilcoxon test. (I) Boxplots showing the fraction X-linked SNPs detected as biallelically expressed per cell in cryo-preserved male E3 and cryo-preserved male E4 embryos (as in main Figure 1D). p value: two-sided Wilcoxon test.

To obtain a transcriptional map of the human preimplantation development, we sequenced the transcriptomes of individual cells isolated from embryos ranging from the 8-cell stage up to the time-point just prior to implantation. After quality control, we retained 1,529 high-quality single-cell transcriptomes from 88 embryos, with an average of 8,500 expressed genes (reads per kilobase of transcript per million mapped reads [RPKM] ≥1; Spearman’s ρ ≥0.63; Figure 1A). A total of 13 to 24 embryos and 81 to 466 cells were analyzed per embryonic day (Figure 1B). To determine the sex of each embryo, we assessed the expression level of Y-linked genes for each cell (Figure S1).

To first study the maternal to zygotic transition, we assessed the activity of ubiquitously expressed Y chromosome genes (i.e., genes exclusively derived from the paternal germline) and found an increase between E3 and E4 (Figure 1C; p = 8.7e−22, Mann-Whitney-Wilcoxon test [MWW]). Furthermore, by detection of single nucleotide polymorphisms (SNPs) in the single-cell RNA-seq reads, we observed that most male E3 cells contained biallelically derived RNA of X chromosome genes (Figure 1D), indicating the presence of lingering maternal transcripts. This biallelic signal was devoid in E4 and later stages (Figures 1D, S1H, and S1I), suggesting that maternal RNA clearance had occurred. Thus, our data point to incomplete zygotic genome activation (ZGA) at E3 that approaches completion by E4, in line with previous studies (Yan et al., 2013).

**Figure S2:**
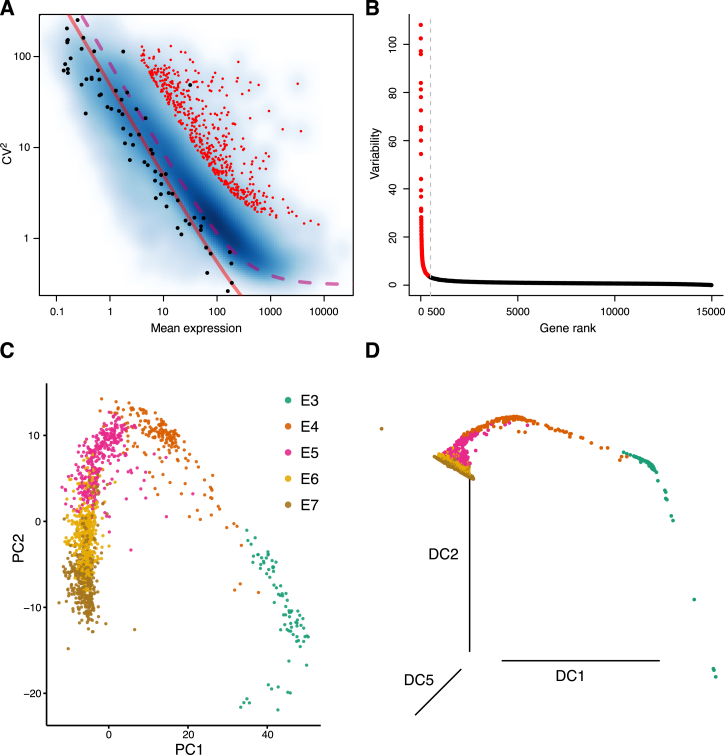
Identification of the Most Variable Genes and Temporal Separation of Preimplantation Single-Cell Transcriptomes, Related to Figure 1 (A) Gene mean expression versus squared coefficient of variation (CV^2^) of all RefSeq genes. Red dots indicate genes ranked as being among the 500 most variable genes. Black dots indicate ERCC spike-in transcripts. Red line represents a fit against the ERCC transcripts, indicating the technical variability, and dotted line represents a biological variability of CV = 0.5, added to the technical one. (B) Variability test-statistic of every expressed RefSeq gene, derived from the mean-variance relationship in Figure S1A (Supplemental Experimental Procedures), versus the gene-rank; where rank was obtained by ordering by the variability test-statistic. Red dots indicate genes ranked as being among the 500 most variable genes. (C) Principal component analysis of all 1,529 cells using the 500 most variable genes. (D) Diffusion map of all 1,529 cells using the 500 most variable genes.

In order to explore the data in an unbiased manner, we carried out dimensionality reduction using the most variable genes across all cells, accounting for the mean-variance relationship present in single-cell RNA-seq gene expression data (Brennecke et al., 2013) (Figures S2A and S2B). We found that regardless of dimensionality reduction technique used, the primary segregating factor was developmental time, as cells were clearly ordered in agreement with embryonic day when projected onto the first dimensionality-reduced components (Figures 1E, S2C, and S2D). To further refine the resolution of the developmental timing of each individual cell, we fitted a principal curve (Hastie and Stuetzle, 1989) to the cells in a t-distributed stochastic neighbor embedding (t-SNE) subspace (van der Maaten and Hinton, 2008) (Figure 1F) and assigned a pseudo-time to each cell based on its projection onto this curve, which we utilized in parts of the temporal analysis.

### Segregation of ICM and TE Appears at E5

**Figure 2:**
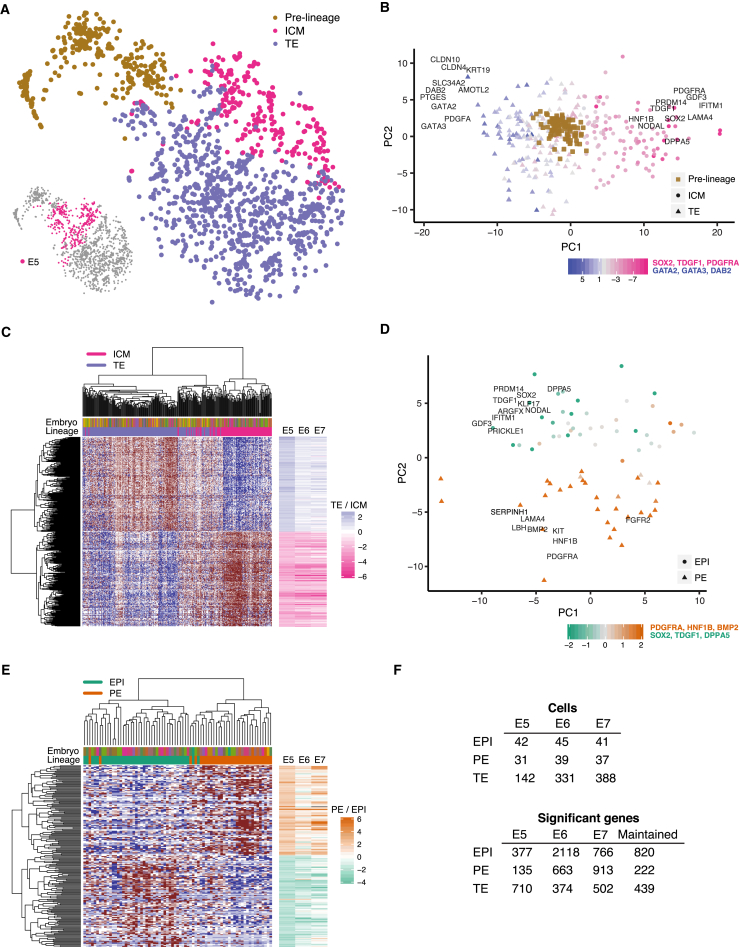
Lineage Segregation of Cells into Inner Cell Mass, Trophectoderm, Epiblast, and Primitive Endoderm (A) t-SNE plot of all cells, as in Figure 1E, showing ICM and TE assignment of cells. Cells from E5 are highlighted in the lower left insert. The ICM-TE cell classification was done using PAM clustering in a PCA dimensionality-reduced sub-space (Figure 2B and Supplemental Experimental Procedures). (B) PCA biplot showing ICM and TE classification of cells from E5. Cells were classified as ICM or TE using PAM clustering in the PCA dimensionality-reduced space with the 250 most variable genes across all non-pre-lineage E5 cells as input (Supplemental Experimental Procedures). Cells in embryos with a pseudo-time <12.5 were assigned as pre-lineage. Genes with high PC loadings are shown. Colors indicate the weighted mean of the expression of previously known lineage markers using weights −1 and 1 for ICM and TE genes, respectively. (C) Heatmap of E5 cells and the top 500 differentially expressed genes between ICM and TE E5 cells (top 250 genes from each lineage). Upper colored bar indicates embryo membership, lower bar indicates lineage. Right-hand-side bars indicate the log2 fold-change of the TE divided by ICM mean-expression level for each gene and embryonic day (E5–E7). (D) PCA biplot showing EPI and PE classification of ICM cells from E5. Cells were classified as EPI or PE using PAM clustering in the PCA dimensionality-reduced space with the 250 most variable genes across all ICM cells that belonged to the right-most hierarchical cell-cluster in Figure 2C (Supplemental Experimental Procedures). Genes with high PC loadings are shown. Colors indicate the weighted mean of the expression of known lineage markers using weights −1 and 1 for EPI and PE genes, respectively. (E) Heatmap of E5 cells and the top 200 differentially expressed genes between EPI and PE E5 cells (top 100 genes from each lineage). Upper colored bar indicates embryo membership, lower bar indicates lineage. Right-hand-side bars indicate the log2 fold-change of the PE divided with EPI mean-expression level for each gene and embryonic day (E5–E7). (F) Number of cells (upper table) and lineage-specific genes (lower table) per embryonic day (E5–E7) and lineage. TE, trophectoderm; EPI, epiblast; PE, primitive endoderm. See also Figure S3 and Tables S1 and S2.

**Figure S3:**
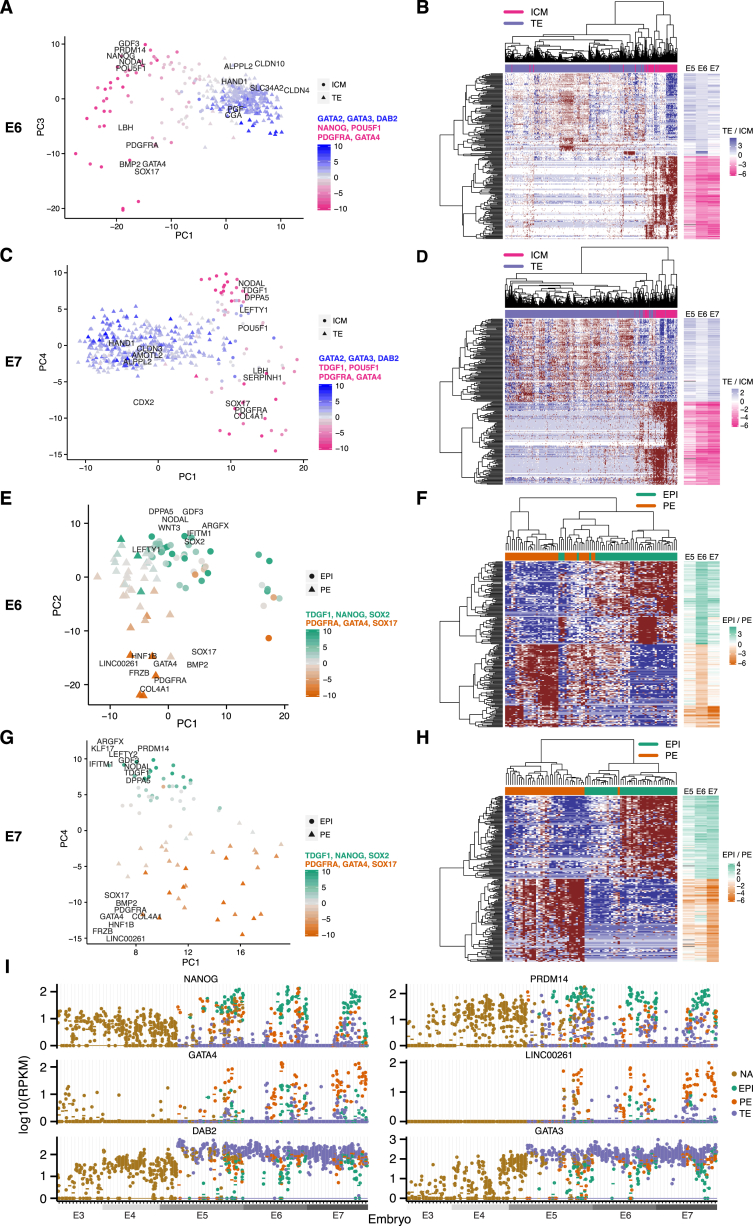
Lineage Segregation of Cells into Inner Cell Mass, Trophectoderm, Epiblast, and Primitive Endoderm, Related to Figure 2 (A) PCA biplot showing ICM and TE classification of cells from E6. Cells were classified as ICM or TE using PAM clustering in the PCA dimensionality-reduced space with the 250 most variable genes across all E6 cells as input (Supplemental Experimental Procedures). Genes with high PC loadings are shown. Colors indicate the weighted mean of the expression of known lineage markers, listed above the color bar, using weights −1 and 1 for ICM and TE genes, respectively. (B) Heatmap of E6 cells and the top 200 differentially expressed genes between ICM and TE E6 cells (top 100 genes from each lineage). The upper colored bar indicates lineage-classification of each cell, as determined in (A). Right-hand-side bars indicate the log2 fold-change of the TE divided with ICM mean-expression level for each gene and embryonic day (E5-E7). (C and D) As in (A) and (B) but with respect to E7 cells. (E and F) As in (A) and (B) but with respect to E6 ICM cells, contrasting EPI and PE cells. (G and H) As in (E) and (F) but with respect to E7 ICM cells, contrasting EPI and PE cells. (I) Expression for a selection of top-ranked lineage-specific marker genes stratified by embryo and lineage. Each dot represents the expression in a single cell and vertical lines segregate embryos. Horizontal lines indicate mean expression level per lineage within each embryo.

The second strongest segregating factor emerged during E5, where the spread between cells sharply increased, perpendicular to the developmental time axis (Figure 2A). This coincided with the time of blastocoel formation, indicating that this time period is critical for the formation of a blastocyst and the emergence of lineages. In order to identify lineages, we applied principal component analyses (PCA) and clustering using the most variable genes (Figure 2B; Supplemental Experimental Procedures). The separation of cells along principal component 1 (PC1) corresponded to the TE and ICM segregation since the genes with the strongest loadings on PC1 were well-known TE lineage markers (*GATA2* and *GATA3*) as well as known ICM markers (*SOX2* and *PDGFRA*). Importantly, these TE and ICM genes were identified as top-genes using an unbiased data-driven approach, starting with 15,633 expressed genes. The same procedure was then applied to E6 and E7 cells to classify the lineage fate of the cells as ICM or TE (Figure S3). Interestingly, applying the same unbiased approach separately to E3, E4, and to only immature E5 cells (those marked as pre-lineage in Figure 2A), no groupings of cells were identified. Similarly, we observed no grouping among these cells when using previously known human and mouse markers (Blakeley et al., 2015, Guo et al., 2010, Yan et al., 2013) nor when using lineage-specific genes identified in this study.

Once cells had been designated as TE or ICM, we performed differential expression analysis between the lineages. The differential expression analysis identified 2,414 genes that were significantly differentially expressed between E5 ICM and TE cells (false discovery rate [FDR] ≤5%); and 2,383 and 3,053 differentially expressed genes in E6 and E7, respectively (Table S1). Selecting the top 500 differentially expressed genes, we found that E5 cells (excluding the immature E5 cells) segregated into three groups (Figure 2C). Two of these groups distinctly expressed either TE or ICM genes in a mutually-exclusive manner, indicating more matured TE and ICM lineages, whereas the third group of cells co-expressed TE and ICM genes but at a lower expression level. Based on this, we denoted the co-expressing cluster of cells as E5.mid (since these cells seemed uncommitted to a particular lineage) and labeled the other two distinct groups as either TE or ICM and denoted them as E5.late. Further, ICM and TE genes identified at E5 tended to maintain their lineage specificity throughout the remainder of the preimplantation development, as their ICM versus TE fold-changes were consistent from E5 to E7, despite that E6 and E7 lineage assignment was done independently of the E5 gene set (right-hand side bars in Figure 2C).

### Segregation of ICM into EPI and PE Appears among E5 ICM Cells

To identify EPI and PE cells, we performed a similar analysis as described above, using the most variable genes within the ICM cells for each embryonic day (Figures 2 and S3). Surprisingly, along the second PC, we found ICM cells as early as E5 separated with respect to EPI and PE lineage-specificity (Figure 2D). Among the genes with the highest PC loadings were pluripotency-related genes and known EPI markers (*SOX2*, *TDGF1*, *DPPA5*, *GDF3*, and *PRDM14*), and among the genes with the most negative PC loadings were genes implicated in endoderm specification (*PDGFRA*, *FGFR2*, *LAMA4*, and *HNF1B*). Differential expression analysis between the EPI and PE cells identified 43, 1,412, and 542 differentially expressed genes at E5, E6, and E7, respectively (FDR ≤5%; Table S1). Furthermore, differentially expressed genes found in E5 maintained their EPI and PE specificity in E6 and E7 (Figure 2E). The number of cells per lineage and embryonic day resulting from the lineage classification is summarized in Figure 2F.

### Lineage-Specific Genes Relate to Cell Fate Functionality

**Figure 3:**
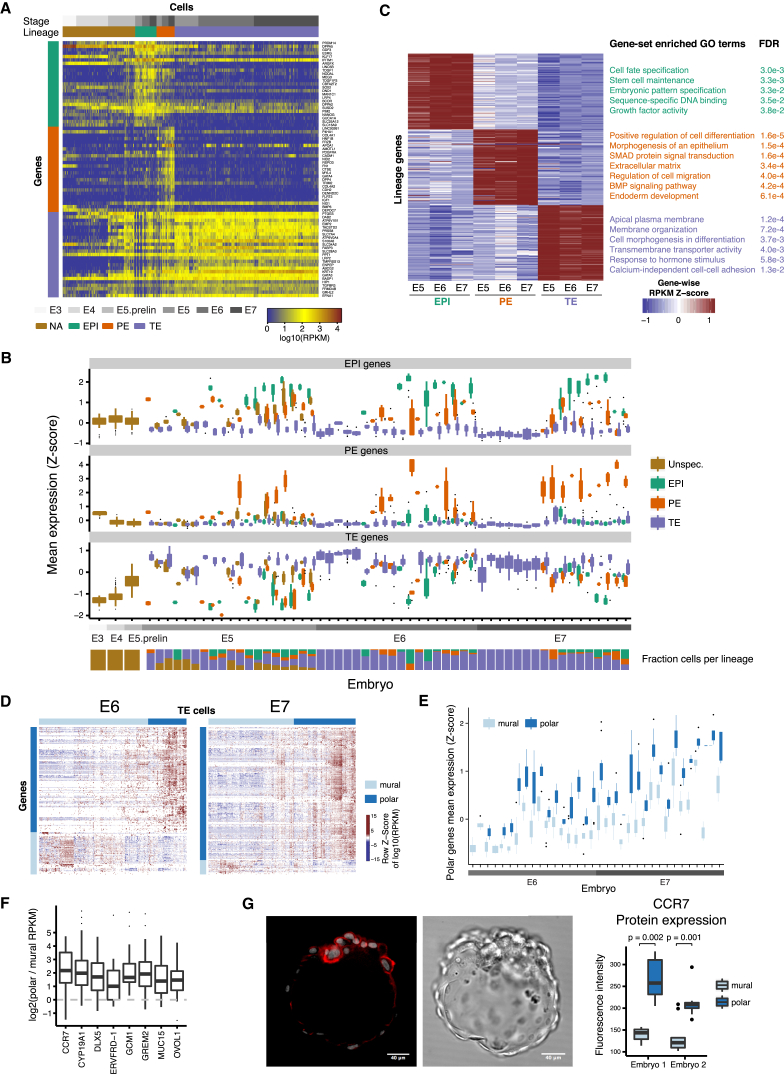
Lineage-Specific Genes Relate to Sub-population Cell Fate (A) RPKM expression heatmap of top 25 maintained (E5–E7) lineage-specific genes, from each lineage, across all cells. (B) Boxplot of mean expression level with respect to top 25 maintained lineage-specific genes, from each lineage, stratified by embryo and lineage. The mean expression across genes was calculated after *Z* score normalization as to account for that genes can be expressed at different scales. (C) Normalized RPKM mean-expression levels and Gene Ontology gene set enrichment results of top 100 lineage-specific genes from each lineage. The mean expression of each gene was calculated per embryonic day and lineage and *Z* score normalized across those strata. (D) Heatmap of top variable genes within TE cells, stratified by embryonic day. Cells were clustered by PAM-clustering in the PC1 and PC2 subspace. Genes were ordered by hierarchical clustering. (E) Boxplot of TE cells with respect to their mean expression level using 129 polar TE genes that were significant in both E6 and E7, stratified by embryo and polar-mural classification. The mean expression across polar-specific TE genes was calculated after *Z* score normalization. (F) Boxplot of polar versus mural expression fold-changes within each embryo. (G) CCR7-stained embryo by immunohistochemistry (IHC) (left). Boxplot of CCR7 IHC fluorescence intensity of polar and mural cells (right; p: MWW p value). See also Tables S3 and S4.

To find lineage-specific genes, we combined the *Z* scores obtained from the differential expression analysis of one lineage against each of the other two (Stouffer’s method; FDR ≤5%; Figure 2F; Table S1). Next, to find genes that maintain their lineage-specificity from E5 to E7, we combined the lineage-specific results across embryonic days, which resulted in 439, 820, and 222 significantly maintained TE-, EPI-, and PE-specific genes, respectively (Stouffer’s method; FDR ≤5%; Figure 2F; Table S2). The top-ranked maintained EPI genes exhibited expression patterns clearly specific for cells of the EPI lineage in E6 and E7 whereas in E5 the EPI genes were to some extent also expressed in PE cells (Figures 3A and 3B). Top-ranked maintained PE genes were specifically expressed across E5 to E7, and TE genes had low expression in E3 and E4 but were expressed in all cells from E5 to E7, although at a higher expression level in TE cells (Figures 3A and 3B). Several known TE markers, such as *GATA3*, *DAB2*, and *GATA2* were among the top-ranked genes (rank 2, 25, and 58, respectively). Interestingly, *CDX2* was differentially expressed, but only ranked 209^th^, and *EOMES* was not expressed at all. In addition to known markers, several less-described markers were identified, such as *PTGES*, *EMP2*, *TGFBR3*, and *PDGFA* (rank 1, 4, 23, and 33). Among top-ranked EPI-specific genes were factors implicated in embryonic preimplantation development in mouse or human, such as *PRDM14*, *GDF3*, *TDGF1*, *NODAL*, *SOX2*, and *NANOG* (rank 1, 3, 9, 10, 12, and 22) and a few less-established markers, including *DPPA5*, *ESRG*, *KLF17*, *ARGFX*, and *DPPA2* (rank 2, 4, 5, 7, 19). PE-specific genes included known factors such as *COL4A1*, *HNF1B*, *PDGFRA*, *GATA4* and *FN1* (rank 3, 4, 7, 13, and 15) and among highly ranked genes were also *LINC00261*, *FRZB*, *AMOTL1*, and *DPP4* (rank 1, 5, 6, and 14). Expression profiles for a subset of the maintained lineage markers are shown for all cells, stratified by embryo, in Figure S3I.

To explore the functional roles of lineage-specific genes, we performed Gene Ontology (GO) gene set enrichment analyses on the top 100 maintained lineage genes from E5 to E7 (Figure 3C; Table S3). EPI-specific genes were enriched for cell fate specification, stem cell maintenance, and embryonic pattern specification. PE-specific genes were enriched for terms such as morphogenesis of an epithelium and endoderm development. TE-specific genes were enriched in apical plasma membrane, cell morphogenesis involved in differentiation, and active transmembrane transporter activity. This is in agreement with the notion that the TE forms an outer layer of cells that acts as a barrier, preventing water and solutes from passing freely through the paracellular space.

### Subpopulations within the TE Lineage

To determine whether subpopulations were present within the lineages, we investigated the most variable genes for each lineage and embryonic day (Supplemental Experimental Procedures). Interestingly, we found two sub-clusters of cells among E6 and E7 TE cells (Figure 3D), and differential gene expression analysis between the two groups of cells (Kharchenko et al., 2014) identified 269 and 349 significantly differentially expressed genes in E6 and E7, respectively (Table S4), of which 135 genes overlapped between E6 and E7 (129 upregulated and 6 downregulated). We identified several genes that have been previously associated with trophoblast differentiation (Figure 3F), including *CCR7* (rank 1) (Drake et al., 2004), *CYP19A1* (rank 4) (Kumar et al., 2013), *DLX5* (rank 5) (Marchand et al., 2011), *ERVFRD-1* (rank 6) (Mi et al., 2000), *GCM1* (rank 7) (Marchand et al., 2011), *GREM2* (rank 8) (Sudheer et al., 2012), *MUC15* (rank 13) (Marchand et al., 2011), and *OVOL1* (rank 16) (Renaud et al., 2015). At an embryo level, we found that the 129 upregulated genes segregated the cells into two clusters consistent with our classification (Figure 3E). These genes were significantly enriched in 38 GO terms, most of which were related to cell-cell signaling including “molecular transducer activity” and “signal transducer activity” (Table S4). The significant terms and genes were consistent with a more differentiated polar subpopulation of the TE cells, relying on cell-cell communication between the endometrium and the implanting polar TE of the blastocyst. Moreover, we observed higher levels of CCR7 protein at the polar side of the embryo (Figure 3G), in both TE and ICM cells, supporting that the identified TE subpopulations likely reflect polar and mural cells.

### Gene Expression-Inferred Developmental Timing Corroborates Concurrent Lineage Segregation

**Figure S4:**
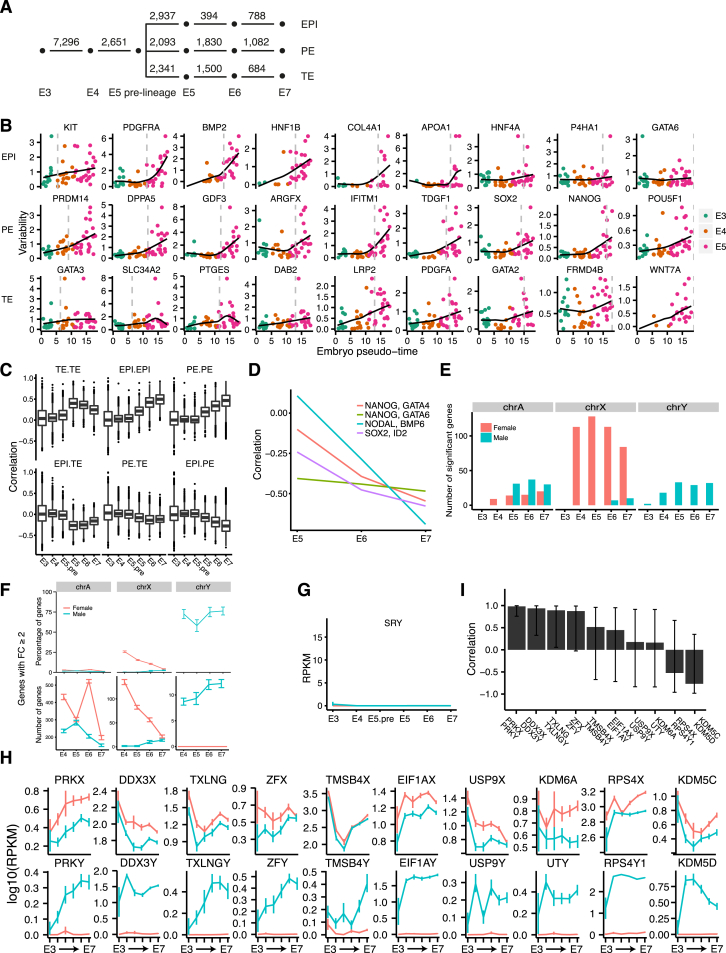
Preimplantation Developmental Progression of Lineage-Specific and Sex-Specific Genes, Related to Figure 4 (A) The number of significantly differentially expressed genes between embryonic time-points. From E5 to E7 the differential expression analysis was done within lineages. (B) Gene expression variability within each embryo versus developmental time (Supplemental Experimental Procedures). Each dot represents an embryo. (C) Gene-gene Pearson correlations among the top 300 maintained lineage genes (100 from each lineage). Titles refer to that genes specifically expressed in each of the two listed lineages were correlated to each other. (D) Pearson correlation within ICM cells against developmental stage for gene-pairs selected among lineage-specific genes with the strongest anti-correlation. (E) The number of significantly differentially expressed genes between females and males at each embryonic day, stratified by the genes’ chromosomal location: autosome (chrA), chromosome X (chrX) and chromosome Y (chrY) (F) The number and percentage of genes with fold-change (FC; female versus male cells) ≥ 2. Error-bars indicate standard deviation obtained by bootstrap resampling of cells (n = 100). Red and blue lines represent genes with higher expression in female and male, respectively. (G) RPKM expression levels of the testis-determining factor *SRY*. (H) RPKM stage-wise mean expression levels of X- and Y-linked paralogous gene pairs that were significantly differentially expressed. Error-bars indicate 95% confidence interval. (I) Pearson correlation between male stage-wise mean expression levels of X- and Y-linked paralogous gene pairs. Error-bars indicate 95% confidence interval.

First, to assess temporal differences we conducted differential gene expression analysis between embryonic time points. In almost every contrast there were more than 1,000 significantly differentially expressed (Figure S4A). Top genes included *DNMT3L* (E3 versus E4), TE genes such as *CLDN4*, *CLDN10*, *GATA2*, and *SLC2A1* (E4 versus E5.pre-lineage) and *CGA* and *PGF*, which were strongly upregulated in all three lineages from E5 to E7 (Table S5).

**Figure 4:**
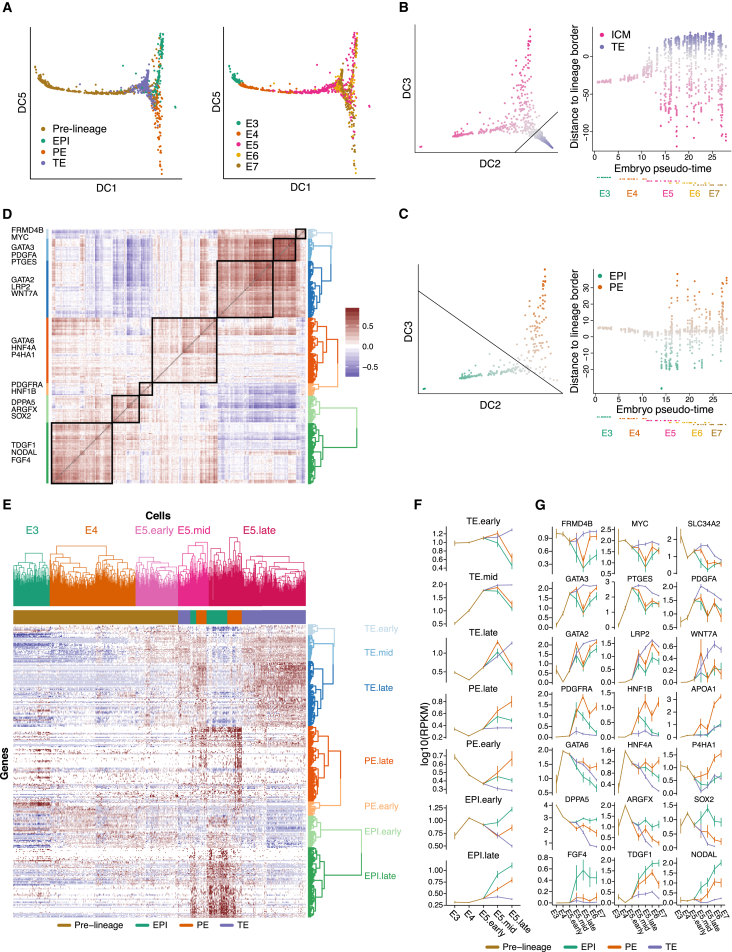
Developmental Progression from E3 to E7 Showing the Formation of Blastocyst Lineages (A) Three-dimensional diffusion map representation of all cells, showing lineage assignment and embryonic day, respectively. A total of 94 lineage-specific genes at E5 were used as input (Supplemental Experimental Procedures). DC, diffusion component. (B) Lineage segregation of all 1,529 cells with respect to ICM versus TE. Left: the expression of every cell with respect to lineage-specific genes (axis represent diffusion-components [DC], analogous to principal components). The black line depicts a lineage-separating border that optimally separates the two classes of cells, determined by a support vector machine (Supplemental Experimental Procedures). Right: the y axis indicates the distance from the lineage decision boundary (black line in the left sub-figure). The x axis indicates pseudo-time, as determined in Figure 1F. Each embryo was assigned a time using the mean of the cellular pseudo-times of the cells in that embryo. Each dot below the x axis indicates an embryo, colored by the embryonic day of sampling. (C) As (B) but with respect to EPI versus PE. (D) Gene-gene Pearson’s correlation matrix using the top 100 lineage-specific genes from each lineage. Gene-modules were determined based on hierarchical clustering of the correlation matrix and labeled with representative genes being part of the cluster. (E) Heatmap of expression levels (RPKM) for E3–E5 cells using the top 100 lineage-specific genes from each lineage. Cell groups were ordered according to their pre-determined groups, indicated by the colored dendrogram, and clustered within their respective group (E3, E4, E5.early, E5.mid, and E5.late). E5.mid cells were classified into three sub-groups based on the observed hierarchical clusters (EPI, PE, and TE). Genes were grouped according to observed hierarchical clusters and named based on which type of cells, and at which time point, the genes were expressed. (F) RPKM mean expression levels of lineage-specific gene sub-clusters as identified in Figure 4D. Vertical lines indicate 95% non-parametric bootstrap confidence interval across cells (B = 1,000). (G) RPKM expression levels of representative genes from each gene sub-cluster. Vertical lines indicate 95% non-parametric bootstrap confidence interval across cells (B = 1,000). See also Figure S4, Tables S5 and S6, and Movie S1.

To obtain a combined view of the lineage specification and developmental state, we applied diffusion map dimensionality reduction (Haghverdi et al., 2015) on all cells using the lineage-specific genes. This revealed the progressive development from E3 to early E5, followed by a split into three lineages (Figure 4A; Movie S1). To further elucidate the dynamics of the lineage specification, we scored the degree of ICM or TE segregation of all cells (as the distance to the ICM-TE decision surface) as a function of inferred developmental time (pseudo-time) (Figure 4B). This corroborated that the blastocyst forms distinct transcriptional states corresponding to lineages during E5, after which the segregation (based on lineage-specific genes) did not further increase. The analyses also revealed that cells of E3 and E4 embryos were more similar to the ICM than the TE, expressing genes that will later become specific to the ICM. We applied the same analysis with respect to the EPI and PE lineages and again observed a separation occurring during E5, which did not increase over time (Figure 4C).

As a complementary approach, we investigated whether individual genes had segregating expression levels before E5. To this end, we calculated a gene expression variability score within each embryo for every gene and regressed it onto embryonic pseudo-time (Supplemental Experimental Procedures). The majority of lineage-specific genes gradually increased in variability and reached their maximum at E5 or later (Figure S4B). Furthermore, lineage-specific genes expressed already during E4 (Figures 4D–4G, described below) also increased in variability at E5 or later, suggesting the existence of a more homogeneous co-expressing state followed by increasingly heterogeneous expression.

### Co-expression of Lineage Markers Precedes Matured Lineages

To investigate the transition from morula to blastocyst in more detail, we focused on cells from E3 to E5 and lineage-specific genes (the top 100 differentially expressed genes in each of the three lineages). The TE-specific genes formed three main clusters (Figures 4D and 4E), reflecting the order at which their expression became on par with that in mature TE cells (denoted TE.early, TE.mid, and TE.late). Also, the PE- and EPI-specific genes formed two main clusters each, corresponding to the time at which they increased in expression levels (Figures 4D and 4E). During E4, the cells tended to express early EPI genes, corresponding to about half of the investigated EPI-specific genes and a smaller subset of PE and TE genes. Interestingly, during early E5 the cells had activated about half of the TE genes (TE.early and TE.mid), while still maintaining the expression of early EPI genes, indicative of an intermediate stage of co-expression of lineage markers. Fewer co-expressing cells were observed at E6 and E7, corroborating that this is indeed a cellular state that precedes maturation of the lineages. The expression dynamics of gene set (Figure 4F) and individual genes (Figure 4G) over embryo stage highlighted that many EPI genes were already turned on in E3 and E4 (e.g., *DPPA5*, *ARGFX*, and *SOX2*), whereas a second group of EPI genes were first turned on in E5.mid, including *FGF4*, *TDGF1*, and *NODAL*.

To extend the gene-dynamics analysis, we calculated pairwise correlations, within each stage, between the top 300 maintained lineage-specific genes (Table S6). Gene pairs from the same lineage drastically increased their correlation in the transitioning from E4 to E5, and within EPI and PE gene sets, the correlations gradually increased from E5 to E7, whereas between TE-specific genes, the correlations decreased in E6 and E7, which may reflect the mural-polar polarization (Figure S4C).

### Preimplantation Sex Differences

To investigate whether sex differences were already present during preimplantation development, we performed differential expression analysis between female and male cells within embryonic day and lineages. We identified 173 differentially expressed genes (FDR ≤5%), out of which 58 were autosomal (0.5% of expressed autosomal genes) (Figures S4E and S4F; Table S7). As expected, *SRY* was not expressed in any cell, indicating that the sex-determination program had not yet initiated (Figure S4G). Thirteen differentially expressed Y chromosome genes were identified, of which nine had X-linked paralogs (Figure S4H). Several of these X-Y paralogous gene pairs had high expression correlations (Figure S4I), suggesting conserved regulation. Strikingly, the X chromosome dominated the contribution of sex-biased genes, having 105 (27% of expressed X genes) significantly higher expressed in female cells but only 7 (1.8% of expressed X genes) higher in male cells, and intriguingly, there was a clear trend of gradual decrease of the female X chromosome overexpression from E4 to E7 (Figure S4F).

### Dosage Compensation of the X Chromosome

**Figure 5:**
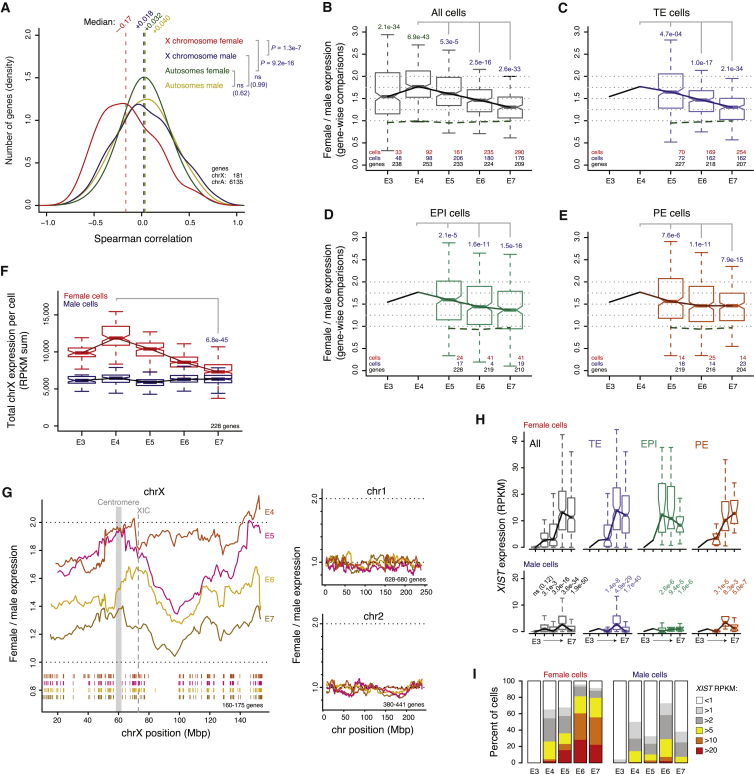
Dosage Compensation of the X Chromosome during Preimplantation Development (A) Distribution of Spearman correlations between gene-expression levels and embryonic day (E4–E7) in female and male cells, for genes located on the X chromosome or autosomes. p values, two-sided MWW. (B–E) Boxplots of female-to-male expression-level ratios of transcribed X chromosome genes, shown for all cells (B) or specific for the TE (C), EPI (D), and PE (E) lineages. Lines intersecting the medians indicate the trend for X chromosome genes, and the green dotted lines around the 1.0-ratio similarly illustrate the medians for autosomal genes. Values above the boxplots denote p values (two-sided MWW), either indicating a significant difference between male and female cells from the same embryonic day (green p values; deviation from one at E3 or E4), or a significant reduction between E4 and a later embryonic day (blue p values). (F) Boxplots showing the distribution of cellular X chromosome RPKM sums for each sex and embryonic day, using a fixed gene set. p value, two-sided MWW. (G) Female-to-male moving expression average along the X chromosome using a 25-nearest-genes window, shown for the stages beyond ZGA completion (E4–E7), and the same for two autosomal chromosomes included for comparison. The ticks below the moving-average lines show the locations of expressed genes included in the estimates, colored according to embryonic day. (H) *XIST* expression-level boxplots per sex, day and lineage. p values indicate significant differences between male and female expression distributions (two-sided MWW; “ns” denotes not significant). (I) The fraction of cells with *XIST* RNA expression above indicated thresholds, stratified by sex and stage. See also Figure S5 and Table S7.

The large number of female and male cells provided the opportunity to evaluate X chromosome expression dynamics throughout human preimplantation. Interestingly, we observed that specifically X chromosome genes tended to become downregulated with time. Spearman correlations between expression level and embryonic time were negative for most X-linked genes in female cells, but not in male cells (Figure 5A; p = 1.3e−7 female versus male, MWW) and not for autosomal genes (p > 0.05). To further study this female-specific downregulation of the X chromosome, we calculated female-to-male relative expression levels for transcribed genes at each embryonic day and cell lineage. This revealed that beyond the completion of ZGA at E4, a stage at which female cells have two active X chromosomes, X-linked genes became gradually dose compensated in all lineages (Figures 5B–5E; p = 4.7e−4 to 2.1e−34, MWW). This equilibration of female and male expression was not a result of transcriptional upregulation in males, since the total X chromosome output per cell remained nearly constant in males but distinctly dropped between E4 and E7 in females (Figure 5F; p = 6.8e−45, MWW). To investigate whether this dampening of female X chromosome expression occurred chromosome-wide, the female-to-male expression was calculated by moving averages along the chromosome. This revealed a gradual and X chromosome-wide dosage compensation mechanism (Figure 5G), with tendency of slightly delayed downregulation of regions around the centromere and the distal q-arm. As expected, autosomes, serving as negative controls, showed equivalent expression in male and female cells (Figure 5G). These data imply that X chromosome-wide dosage compensation takes place in all three cell lineages, initiating between E4 and E5 and reaching an overall ∼70%–85% compensation at E7. This is dependent on chromosomal region and whether expression-ratios of individual genes (Figures 5B–5E) or the total X chromosome expression output (Figure 5F) is considered.

### *XIST* and *XACT* Expression

**Figure S5:**
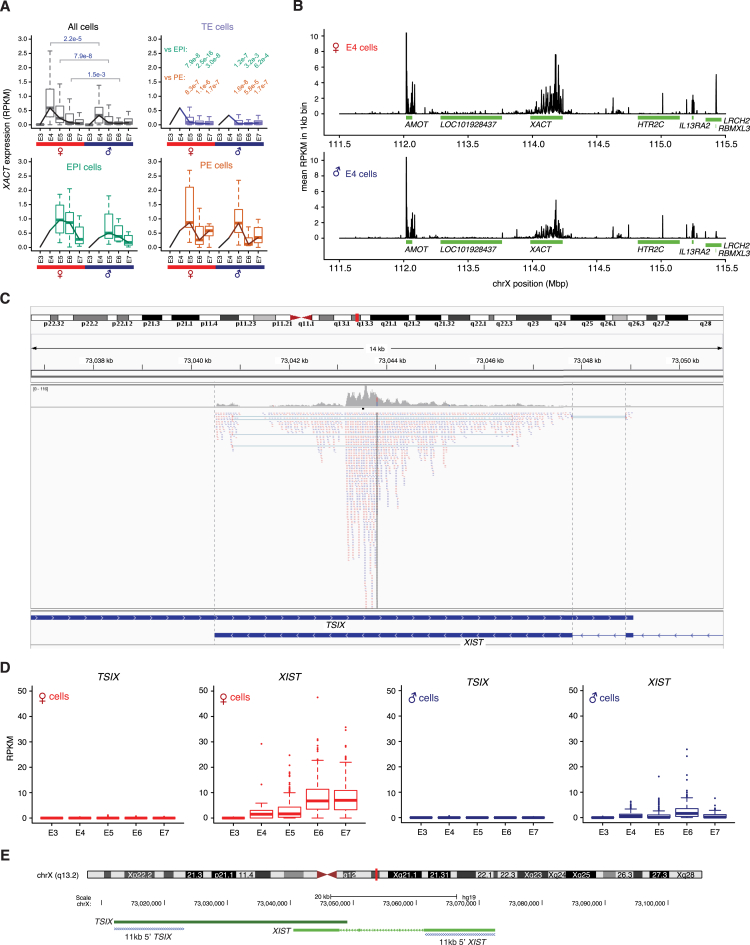
Detection of *XACT* and *XIST* RNA in Human Preimplantation Cells, Related to Figure 5 (A) *XACT* lncRNA expression-level boxplots per sex and lineage. p values were derived from comparing the expression distributions (two-sided Wilcoxon test). (B) Barplots showing the average expression level (RPKM) within 1kb bins along a segment of the X chromosome, for female and male E4 cells. A broad peak of mapped reads appear at the *XACT*-gene sequence (chrX:112,983,323-113,235,148). (C) Mapped sequence reads, from a female E7 cell, aligned to the genomic region where *XIST* (minus strand) and *TSIX* (plus strand) overlap. This shows the lack of *TSIX*-mapping reads (no reads in *TSIX*-unique segments) as well as the biallelic expression of an *XIST* SNP (marked as a red-blue bar). (D) Expression-level (RPKM) boxplots for *XIST* and *TSIX* in male and female cells (including all cells and embryonic days), calculated from two non-overlapping *XIST* and *TSIX* sequences corresponding in length (11 kb 5′ sequence of each gene). *XIST* had 431-fold higher expression than *TSIX* at stage E7, indicating that the biallelic detection of the *XIST* SNP was not *TSIX-*derived. (E) Exon-intron structure of human *XIST* and *TSIX*, with the 11 kb 5′ sequences used in (D) indicated.

Interestingly, X chromosome dosage compensation coincided with an upregulation of *XIST* in female cells (Figures 5H and 5I). We also detected sporadic *XIST* expression in male cells, although at substantially (∼15-fold) lower levels (Figure 5H; p = 3.1e−3 to 1.9e−50, MWW). Transcription of *XACT,* an X-linked lncRNA recently shown to cover *XIST*-free X chromosomes in cultured human embryonic stem cells (hESCs) (Vallot et al., 2015), was activated at E4 in both sexes, but at significantly higher levels in females (Figures S5A and S5B; p = 2.2e−5, female versus male at E4). Moreover, *XACT* expression was reduced in TE cells already at E5, while its expression level was maintained slightly longer in EPI and PE cells.

### Biallelic Expression of Dose-Compensated Genes

**Figure 6:**
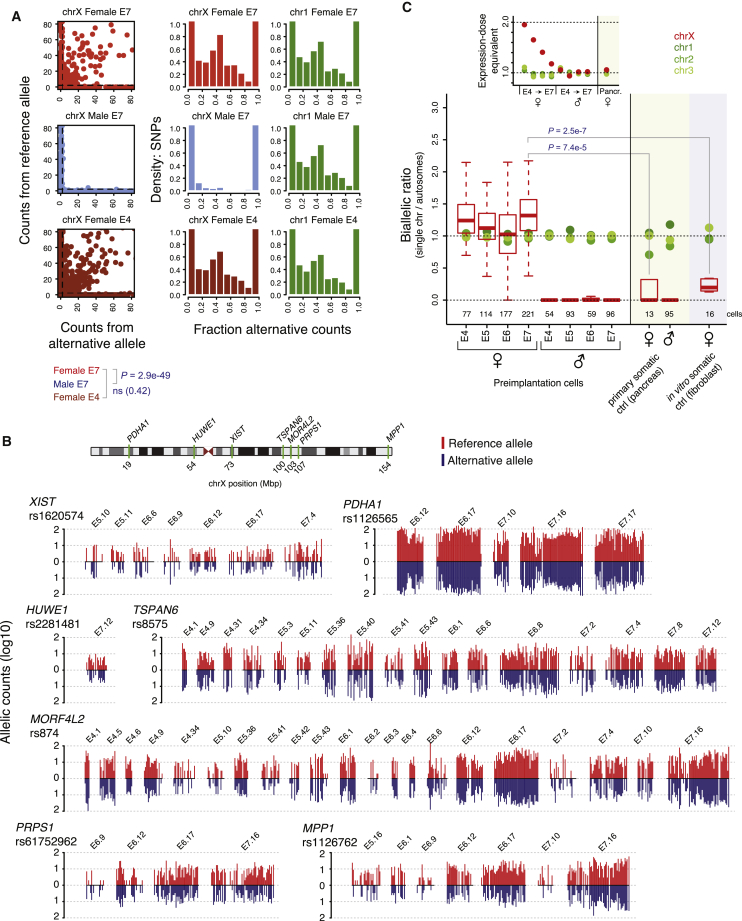
Biallelic Expression of *XIST* and X-linked Genes (A) Scatterplots showing allelic expression levels with the number of reads aligned to the reference and alternative allele on the y and x axis, respectively (shown for 30 random cells from E7 or E4). SNVs with monoallelic expression lie along the axes. Histograms summarize the observed allelic expression ratios of all X chromosome SNVs over all cells, grouped by sex and embryonic day. Chromosome 1 histograms are included for comparison. (B) Allele-specific expression barplots per cell, grouped by embryo, showing the number of reads aligned to the reference and alternative allele, using all female embryos carrying the indicated SNP. Data for a SNP within *XIST*, as well as SNPs located within six other X-linked genes are shown. Cells without any bar lacked reads spanning the SNP position. Biallelic expression in E7 cells was confirmed for these genes by Sanger sequencing (Figures S6A–S6D). (C) Boxplots showing the proportion of biallelic expression from the X chromosome (chrX) relative to that of autosomes (fraction biallelic chrX SNVs / fraction biallelic autosomal SNVs), shown for female and male E4–E7. Human primary pancreatic alpha cells and in vitro female fibroblasts are included as a control reference, representing somatic cells with conventional XCI. Green dots indicate medians when performing the same analysis on individual autosomal chromosomes (shown for chr1-3). Cells with at least 25 detected chrX SNPs were considered. The panel above the boxplots, “Expression-dose equivalent,” indicates the female-to-male total X chromosome-wide expression dose (median ratio of total expression in Figures 5F and S6G) for stages and cell types for which both female and male data were available (E4 to E7 and pancreatic cells), and the same for chr1-3. See also Figure S6.

**Figure S6:**
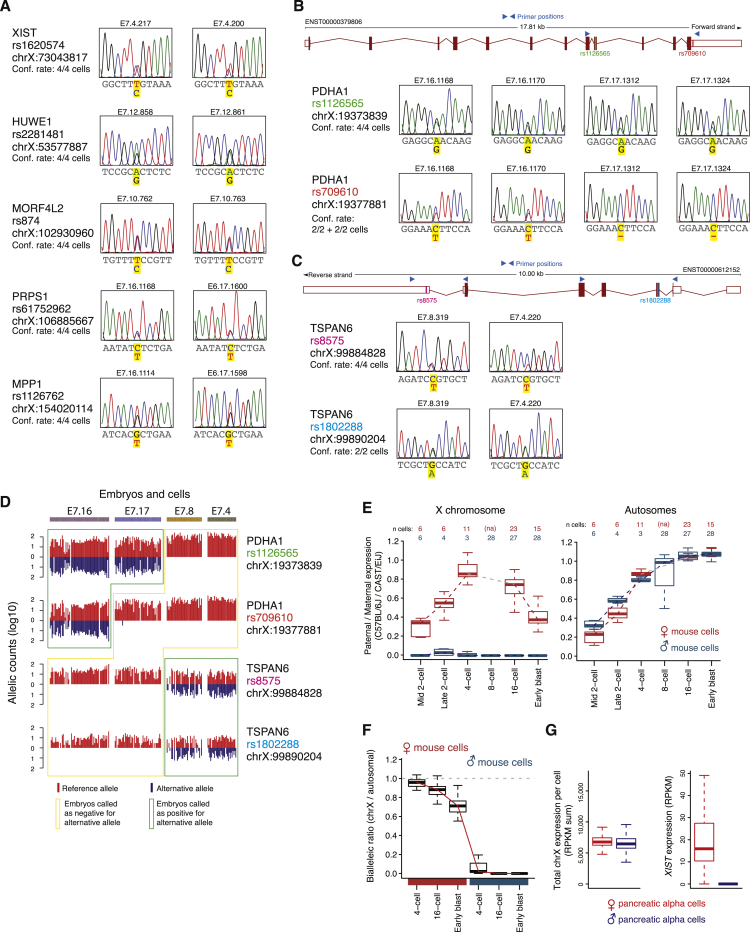
Control Experiments for Allelic Detections, Related to Figure 6 (A) To validate the accuracy of the RNA-seq data SNP calling, and to confirm the biallelic X chromosome expression with an alternative detection method, we performed Sanger sequencing on amplicons from female E7 cDNA libraries. This analysis was performed for each of the SNPs and genes presented in Figure 6B, using 4 separate single-cell libraries per gene. All of the cDNA libraries tested by the Sanger sequencing were confirmed to be biallelic for the evaluated SNP (“Conf. rate”), and the chromatograms for two example cells are shown for each SNP. The code above each chromatogram denotes the cell ID (embryonic day, embryo ID, cell ID). (B) We further evaluated two SNPs located within the same gene (*PDHA1*) and PCR amplicon, for which one embryo had heterozygous expression at both SNPs and another embryo had heterozygous expression at only one of the two SNPs according to our single-cell RNA-seq data (allelic RNA-seq data for these embryos is shown in D). The Sanger sequencing confirmed this pattern. (C) For one gene, *TSPAN6*, we additionally generated two separate amplicons for Sanger sequencing. Cells that had heterozygous expression for the two different SNPs located within these disjoint amplicons (according to the single-cell RNA-seq data, shown in D) were also confirmed to have heterozygous expression at both SNPs by Sanger sequencing. (D) Allele-expression barplots (as in main Figure 6B) shown for embryos from which single-cells were used for the double Sanger validations presented in (B) and (C). (E) Allele-level expression boxplots of mouse preimplantation cells from different stages, showing the paternal (C57BL/6J) / maternal (CAST/EiJ) expression ratio per cell on the y axis. This indicates that paternal X chromosome inactivation reached ∼60% completion at the mouse early blastocyst stage. The plots in (E) represent a re-analysis of our previously reported data (Deng et. al, 2014a), but using the same threshold for calling monoallelic expression as used in the current study (Supplemental Experimental Procedures). (F) Boxplots of allele-resolved mouse expression data, showing the ratio of biallelic expression of chromosome X relative to that of autosomes (fraction biallelic chrX SNPs / fraction biallelic autosomal SNPs), at different stages following the zygotic genome activation. (G) Boxplots showing the distribution of cellular X chromosome RPKM sums for female and male primary pancreatic alpha cells, used as positive control for conventional XCI. This indicates that the X chromosome dose is balanced in these somatic cells cells, as expected due to XCI. (H) Expression-level boxplots of *XIST* in female and male primary pancreatic alpha cells, used as positive control for conventional XCI.

To investigate whether the observed dosage compensation process possessed hallmarks of XCI, we sought to investigate the X chromosome expression at an allelic resolution. Although parental allelic origin was not available, we could call the allelic expression for each single nucleotide variant (SNV) present in the Single Nucleotide Polymorphism Database (dbSNP) (Sherry et al., 2001) within each cell, as either undetected, biallelic, or monoallelic for the reference or alternative allele (Supplemental Experimental Procedures). Surprisingly, the degree of biallelic X chromosome expression in female E7 cells was similar to that of female E4 cells, in which two X:es are active (Figure 6A; p > 0.05, female E4 versus E7, Fisher’s exact test). The low frequency of biallelic X chromosome SNVs in male cells verified the accuracy in the allelic expression analysis (Figure 6A; p = 2.9e−49, male E7 versus female E7, Fisher’s exact test). Furthermore, embryos carrying a SNP within the *XIST* gene showed that it was biallelically expressed throughout the progression of dosage compensation (Figures 6B and S5C–S5E). Biallelic expression was also observed for individual X-linked genes that are normally subjected to conventional XCI in mature tissues, even at E7 (Figure 6B). To validate the SNP calls and biallelic expression of X chromosome genes in female E7 cells, we Sanger-sequenced SNP-containing sequences from the single-cell cDNA libraries, indeed confirming the allelic pattern of 36/36 tested samples or SNPs (Figures S6A–S6D).

Moving beyond single-gene analyses, we assessed whether the X chromosome as a whole progressed toward more monoallelic expression during female preimplantation development. To do this, we determined the fraction of biallelic and monoallelic expression for chromosome X, as well as for autosomes in each cell. Monoallelic detection using single-cell RNA-seq can appear both due to transcriptional bursting as well as from technical dropout of RNA molecules (Reinius and Sandberg, 2015), but regulated monoallelic expression such as that of gradual XCI is readily detectable (Deng et al., 2014a). Under a conventional model of XCI (i.e., a single X chromosome becoming inactivated), we therefore expected the fraction of biallelic detections from the X chromosome to steadily decrease between E4 and E7 in female cells. In contrast, we found that the X chromosome’s biallelic fraction did not decrease as the dose equilibration progressed, but remained similar to that of autosomes (Figure 6C). This pattern contrasted markedly with the decreased biallelic fraction observed in mouse (Figures S6E and S6F), utilized as a positive control for validation of the approach, in which ∼60% X inactivation is reached by the early blastocyst stage. As control of completed conventional XCI in human, we analyzed single-cell RNA-seq libraries from primary pancreatic alpha cells, which displayed female-to-male dosage compensation of X chromosome-wide expression as expected (Figure S6G). As an additional control, we analyzed in vitro cultured human female fibroblasts. Both of these somatic cell types showed lowered rates of biallelic expression compared to female E7 preimplantation cells (p = 7.4e−5 and 2.5e−7, MWW; Figure 6C), consistent with the inactivation of one X chromosome in the somatic cells, but not in E7 preimplantation cells.

### Dual *XIST* Clouds with Biallelic Expression of *ATRX*

**Figure 7:**
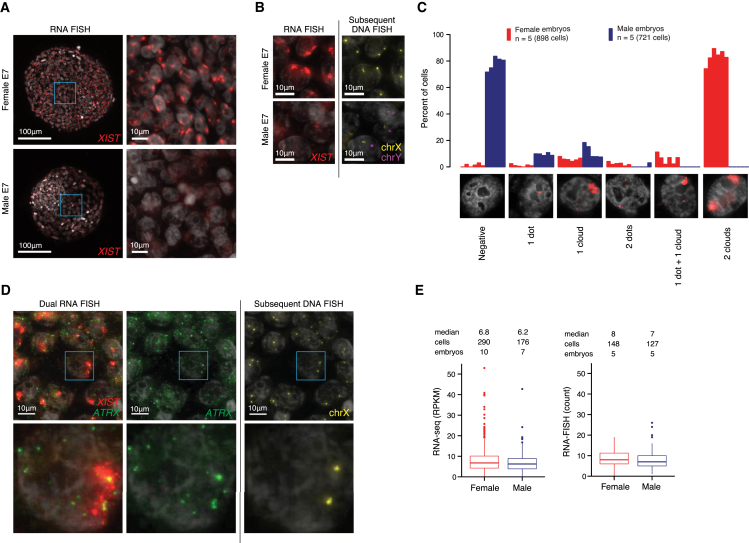
Single-Molecule RNA-FISH Confirmed Biallelic Expression of *XIST* and *ATRX* (A) Single-molecule RNA-FISH of *XIST* shown for a female and male E7 embryo. Zoomed-in regions (right) highlight that two *XIST* clouds (red) were observed in female nuclei (white, Hoechst-stained), but not in male. (B) *XIST* clouds were localized at the X chromosomes (sex chromosomes were identified via DNA-FISH, staining chrX:p11.1–q11.1). (C) Barplot with RNA-FISH *XIST* count statistics from 898 female cells (five embryos) and 721 male cells (five embryos), categorized by the *XIST* localization pattern observed in the nucleus. (D) Left: single-molecule RNA-FISH of *ATRX* and *XIST* in a female E7 embryo. Two stronger ATRX speckles were typically observed within the nuclei, positioned at the *XIST* clouds. Right: DNA-FISH of chromosome X, indicating that the two stronger nuclear *ATRX* dots localized to the X chromosomes. (E) Boxplots of E7 RNA-seq and RNA-FISH *ATRX* expression levels. RNA-FISH counts confirmed that the expression levels of *ATRX* in female and male were on par (mean 8.9 and 8.0; median 8 and 7, respectively), indicating dosage compensation at E7.

We analyzed the localization and allelic expression pattern of *XIST* in female (n = 5) and male (n = 5) E7 embryos by strand-specific single-molecule RNA FISH. The majority of female cells (mean 83%) had dual *XIST* coats and an additional ∼6% of cells displayed biallelic expression with skewed coating (Figures 7A–7C), and only ∼6% of cells had one *XIST* coat. In contrast, ∼11% of male cells had an *XIST* coat while ∼78% of the male cells were *XIST*-negative (Figure 7C). In parallel to *XIST*, we included RNA probes for the X-linked gene *ATRX* (Figure 7D), which is dosage compensated at E7 (female-to-male fold-change 1.08 at E7 p > 0.05; 2.01 at E4 p = 5.4e−8, MWW). Nascent-located dots indicated that *ATRX* was biallelically expressed in female cells with dual *XIST* coats (Figure 7D). To verify that *ATRX* was dosage compensated, we blindly counted single-molecule *ATRX* specks in female and male cells. This confirmed dosage compensation of *ATRX* at E7 (median 8 and 7 molecules per cell count area in female and male respectively, fold-change = 1.14, p > 0.05) (Figure 7E). Altogether, our single-cell RNA-seq and RNA FISH data suggest that X chromosome dosage compensation in the human preimplantation embryo is accomplished by reducing the expression of both X chromosomes, in contrast to the complete silencing of one randomly selected X chromosome that occurs later in development.

## Discussion

We generated a transcriptional resource of human preimplantation development including 1,529 individual cells from 88 embryos. The inclusion of a large number of embryos per stage will dilute out embryo-specific differences that might arise due to embryo-specific genetic variation and abnormalities. Indeed, the analyses of the complete dataset revealed that cellular transcriptomes primarily segregated according to embryonic stage, followed by segregations into lineages (TE-ICM and EPI-PE), embryo-to-embryo variability and subpopulations (polar to mural TE).

Our analyses demonstrated that the segregation of all three lineages occurs simultaneously, given our temporal resolution, and coincides with blastocyst formation at E5. This is in contrast to the model developed from mouse studies where the TE and ICM fate is initiated in a positional and cell polarization-dependent manner within the morula (Cockburn and Rossant, 2010), followed by a subsequent progressive maturation of EPI and PE that is driven by Fgf signaling in the blastocyst (Yamanaka et al., 2010). As human morula compaction occurs at the 16- and not the 8-cell stage (Nikas et al., 1996), a delay in lineage segregation is not entirely surprising and this observation is also in agreement with a previous paper showing CDX2 expression only in the expanded human blastocyst (Niakan and Eggan, 2013). It should also be noted that human compaction is not as prominent as in the mouse, with partial compaction occurring in some blastomeres, further delaying the formation of distinct inner-outer compartments. In the late E4 compacting morula cells, a transcriptional TE program is initiated, including increased expression of *GATA3*, *PTGES*, and *PDGFA*. Importantly, this transcriptional induction occurs while simultaneously co-expressing EPI and PE genes. It is not until E5, during blastocyst formation, that these co-expressed lineage genes start to become mutually restrictive.

In addition to elucidating the dynamics of lineage specification, our analyses identified novel and less-studied genes that may be important for preimplantation development. For example *ARGFX*, ranked as the seventh most EPI-specific gene, is a proposed homeobox gene where the coding region is disrupted in most mammalian genomes analyzed, with exception of human (Li and Holland, 2010). *LINC00261*, the top ranked gene enriched in PE, was recently identified as a definitive endoderm-specific lncRNA driving *FOXA2* expression through recruitment of SMAD2/3 to its promoter (Jiang et al., 2015). With *LINC00261* and *FOXA2* being ranked as number 1 and 34 among the PE-specific transcripts, it is reasonable to speculate that this lncRNA may be an important regulator of PE specification.

The extensive dataset we present here revealed that gradual dosage compensation of the X chromosome occurred in all three lineages during human preimplantation development with both X copies still being actively transcribed throughout this process. Further, the biallelic expression of *XIST* and other X-linked genes in E7 blastomeres are consistent with the patterns of nascent RNA stains previously obtained by RNA-FISH (Okamoto et al., 2011) although conclusions derived solely from the allelic patterns in these earlier studies may have led to an opposite stand regarding the occurrence of dose compensation. Studies on cultured human ESCs have generated rather divergent observations regarding their XCI status (Lessing et al., 2013), and our data suggest that the human pluripotent ground-state should be characterized by female cells expressing *XIST* and having both X chromosomes active while still demonstrating female to male dosage compensation.

The issue of unequal sex-chromosome dose has both emerged and been resolved many times during evolution, using diverse strategies (Deng et al., 2014b, Mank, 2009). Even between mammalian taxa, there exists separate solutions to dosage compensation (Escamilla-Del-Arenal et al., 2011), and *XIST* is an exclusively eutherian invention. Intriguingly, the conventional XCI model where one of the two X chromosomes is inactivated, as demonstrated in the mouse (Mak et al., 2004, Okamoto et al., 2005), does not satisfactorily explain the dynamics of X chromosome expression we observed in human preimplantation development. Instead, the data fit better with a model of an initially dual and partial expression dampening of the two X chromosomes. *XIST* represents an obvious candidate as a mediator for this dampening. However, the possibility that another system, conceivably the evolutionary traces of a more ancient dosage compensation mechanism, might act as a second layer of compensation in human preimplantation development should also be considered.

Finally, the transcriptional atlas of the human preimplantation embryo we provide here has unprecedented cellular and temporal resolution and will therefore be a unique resource in future research aiming to better understand human development and embryonic stem cells.

## Experimental Procedures

Human embryos were obtained from two cohorts at the Huddinge Karolinska Hospital and Carl von Linné Clinic with ethical approval from regional ethics board (2012/1765-31/1). The first cohort was from preimplantation genetic diagnosis (PGD) testing on embryonic day (E) 4 and cultured until E7 (expanded blastocyst, just prior to implantation) under standard conditions as performed in the IVF Clinic (5% CO_2_/5% O_2_ in CCM media (Vitrolife) covered with Ovoil (Vitrolife). The second cohort was from frozen E2 embryos thawed (ThawKit Cleave, VitroLife) and cultured in G-1 Plus media (VitroLife) and from E3 in CCM media. As we are restricted to embryos cultured in vitro, we cannot exclude potential differences with their in vivo counterparts. However, we anticipate these differences to be relatively subtle as in vitro cultured embryos used in infertility treatment progress and give rise to viable offspring.

Embryos were dissociated through trituration in TrypLE, (Life Technologies) and picked with fine glass capillaries. For a subset of E5–E7 embryos, ICM cells were enriched using immunosurgery (15 embryos). Cells were dispensed in lysis buffer, and cDNA libraries were generated using Smart-seq2 (Picelli et al., 2014). Briefly, following cell lysis, PolyA(+) RNA was reverse transcribed using SuperScript II reverse transcriptase (Invitrogen) and nested primers, utilizing a strand-switch reaction to add a reverse primer for the second-strand synthesis. The cDNA was amplified by PCR (18 cycles) using KAPA HiFi HotStart ReadyMix (KAPA Biosystems) and purified using magnetic beads. The quantity and quality of the cDNA libraries were assessed using an Agilent 2100 BioAnalyzer (Agilent Technologies). cDNA (∼1 ng) was tagmented using transposase Tn5 and amplified with a dual-index (i7 and i5; Illumina; 10 cycles) and individual Nextera XT libraries were purified with magnetic beads. Indexed sequence libraries were pooled for multiplexing (∼40 samples per lane), and single-end sequencing was performed on HiSeq 2000 using TrueSeq dual-index sequencing primers (Illumina). For further details and data analysis see the Supplemental Experimental Procedures.

## Author Contributions

R.S. and F.L. conceived the study. S.P., Q.D., S.P.P., A.P.R., and F.L. performed embryo experiments. D.E. and B.R. performed computational experiments. S.C. and S.L. assisted in the RNA-FISH analysis. S.P., D.E., B.R., R.S., and F.L. interpreted data and wrote the manuscript.

## Accession Numbers

The accession number for the raw read sequence data, cell annotations, and RPKM and read count expression matrices for all cells reported in this paper is ArrayExpress: E-MTAB-3929.

## Supplemental Information

Document S1. Supplemental Experimental Procedures

Table S1. Differential Gene Expression Analysis of Preimplantation Lineage Differences, Related to Figure 2 Differential expression test-statistica per lineage and embryonic day, for all RefSeq genes with respect to contrasting lineages.

Table S2. Maintained Lineage Genes from E5 to E7, Related to Figure 2 List of 300 maintained lineage-specific genes (100 genes from each of the lineages, EPI, PE, and TE) and their mean expression levels per lineage and embryonic day. These genes were top-ranked with respect to tending to maintain their lineage specificity from E5 to E7 and were used for GO analysis (Figure 2) and for developmental progression analysis (Figure 4).

Table S3. GO Analysis of Maintained Lineage Genes, Related to Figure 3 Gene set enrichment analysis of Gene Ontology terms with respect to 300 maintained lineage genes (Table S2).

Table S4. Differential Gene Expression and Gene Ontology Analysis of TE Subpopulation Differences, Related to Figure 3 Differential expression test-statistica per sub-population and embryonic day (E6 and E7), for all RefSeq genes, with respect to contrasting TE sub-populations. Rightmost columns contain Gene Ontology gene set enrichment analysis results of 129 polar genes (Supplemental Experimental Procedures).

Table S5. Differential Gene Expression Analysis of Embryonic Day Differences, Related to Figure 4 Differential expression test-statistica per embryonic day and lineage, for all RefSeq genes, with respect to contrasting embryonic days.

Table S6. Gene-Gene Pearson Correlations, Related to Figure 4 Correlations were calculated amongst the top 300 maintained lineage genes (100 from each lineage).

Table S7. Differential Gene Expression Analysis of Preimplantation Sex Differences, Related to Figure 5 Differential expression test-statistica per lineage and embryonic day, for all RefSeq genes with respect to contrasting cells by sex.

Movie S1. Preimplantation Temporal Progression and Lineage Segregation, Related to Figure 4 Three-dimensional diffusion map representation of all cells using 94 lineage-specific genes at E5 as input.

Document S2. Article plus Supplemental Information
