# Single-Cell RNA-Seq Reveals Lineage and X Chromosome Dynamics in Human Preimplantation Embryos

**DOI:** 10.1016/j.cell.2016.08.009

**Published:** 2016-09-22

**Authors:** Sophie Petropoulos, Daniel Edsgärd, Björn Reinius, Qiaolin Deng, Sarita Pauliina Panula, Simone Codeluppi, Alvaro Plaza Reyes, Sten Linnarsson, Rickard Sandberg, Fredrik Lanner

(Cell *165*, 1012–1026; May 5, 2016)

Several errors occurred during the preparation of the above article analyzing the transcriptomes of single cells from human embryos at embryonic days (E) 3–7 and describing the segregation of prelineage (prelin) cells into trophectoderm (TE), primitive endoderm (PE), and epiblast (EPI) and X chromosome dynamics.

We generated additional E3 and E4 cell data during revision that appeared within figures in the published text but were inadvertently omitted from Tables S2, S5, and S7. These tables have now been updated. To reflect the updated Table S7, the number of significantly differentially expressed genes between embryonic time points has also been updated in Figure S4A, as shown below.

In Table S1, the subheadings of the mean and standard deviation columns were mislabeled as EPI and PE throughout the spreadsheet, even where the data presented were derived from other lineages. The correct column headings appear in the updated Table S1.

In Table S5, we accidently inverted the prefixes “E5” and “E5_prelin” in labels of columns containing mean expression and standard deviation in the three sections with the subheadings “E5_prelinvsE5_EPI”, “E5_prelinvsE5_PE” and “E5_prelinvsE5_TE.” In addition, in sections comparing E5 vs E6 and E6 vs E7 cells in Table S5, we presented the data by lineage first and then time (EPI [E5vsE6, E6vsE7], PE [E5vsE6, E6vsE7], and TE [E5vsE6, E6vsE7]). However, the labels of these columns appeared as time first and then lineage (E5vsE6 [EPI, PE, TE] and E6vsE7 [EPI, PE, TE]), resulting in a mismatch between the label and the data columns. These have now been corrected.

None of these changes, which appear in the article online, affect the conclusions of the article. We sincerely regret these mistakes and apologize for any confusion that may have arisen.

**Figure:**
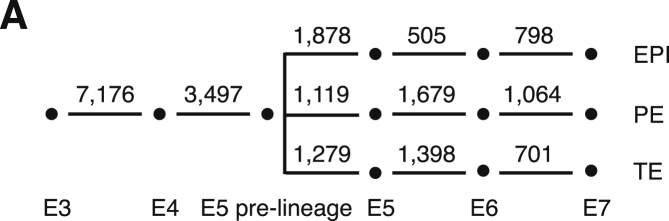
Figure S4A. Preimplantation Developmental Progression of Lineage-Specific and Sex-Specific Genes, Related to Figure 4 (corrected)

**Figure:**
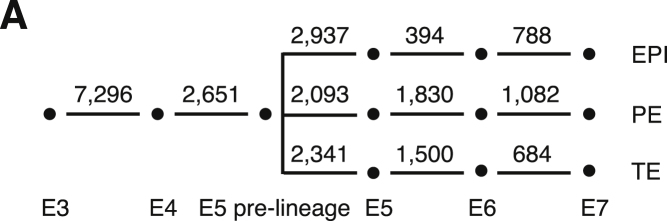
Figure S4A. Preimplantation Developmental Progression of Lineage-Specific and Sex-Specific Genes, Related to Figure 4 (original)

