# Supplementary material for: Single-Cell RNA-Seq Reveals Lineage and X Chromosome Dynamics in Human Preimplantation Embryos
Source: Cell. 2016 May 5;165(4):1012–26. doi: 10.1016/j.cell.2016.03.023 (PMC4868821; doi:10.1016/j.cell.2016.03.023)
Supplement: Document S1. Supplemental Experimental Procedures [file mmc1.pdf]

Cell, Volume *165*

Supplemental Information

Single-Cell RNA-Seq Reveals Lineage and X Chromosome Dynamics in Human Preimplantation Embryos

Sophie Petropoulos, Daniel Edsgärd, Björn Reinius, Qiaolin Deng, Sarita Pauliina Panula, Simone Codeluppi, Alvaro Plaza Reyes, Sten Linnarsson, Rickard Sandberg, and Fredrik Lanner

Supplemental Experimental Procedures

Immunosurgery

For immunosurgery, 1 droplet (30μl) of anti-human antibody (1:3 dilution, Sigma-Aldrich H8765) was aliquoted per embryo onto pre-warmed plastic dishes. Zona pellucidae were removed with Tyrode's solution (Sigma-Aldrich) and placed into droplet for at least one hour in incubator. Embryos were then washed 3x with KSOM medium (Millipore) and subsequently placed into a droplet containing complement guinea pig sera (Sigma-Aldrich; S1639) for 15 mins. Thereafter, embryos were placed into another droplet of KSOM medium and returned to the incubator for 30 mins-1 hour and periodically triturated to isolate the ICM. Once the ICM had been isolated, it was processed as described above.

Fibroblasts

Commercially available normal human dermal fibroblasts (CC-2511, Lonza) generated from a Caucasian female (31 years old) were utilized as a control for the X-dosage compensation analysis. Cells were cultured (DMEM, 10% FBS, 50U/ml 50mg/ml penicillin-streptomycin; Life Technologies) until passage 12, at which time they were picked and processed as described in the main text for embryo cells.

Immunostaining

Embryos were fixed (4% formaldehyde) immediately upon removal from incubator, permeabilized (0.3% Triton-X-100 in PBS) and then blocked with blocking solution (0.1% Tween20 and 4% FBS (10082-147; ThermoFisher) in PBS) for 4 hours at room temperature. Embryos were then incubated in primary antibody overnight at 4°C, followed by 3x5 mins washes in blocking solution and placed in secondary antibody, AlexaFluor 555 donkey anti-rabbit (1:1000 in blocking solution; A31572; LifeTechnologies) for 2hrs, followed by Hoechst 33342 (1μg/ml; LifeTechnologies) nuclear stain for 20 mins at room temperature. All incubations and washes were carried out in a clean well using 4 well plates (734-2176, VWR). Embryos were mounted on a SuperFrost/Plus slide with spacers filled with 17μl of PBS. Embryos were imaged on an Andor spinning disk confocal microscope with 20x dry objective, using an Andor EM-CCD camera. Z-stack images where then processed using IMARIS (Bitplane) and ImageJ (http://imagej.nih.gov/ij/).

RNA FISH

E7 embryos were fixed with 4% formaldehyde (Polysciences) for 15 mins at room temperature, followed by a wash with PBS. Fixed embryos were placed on a silanized glass coverslip and dried for approximately 2 mins. Coverslip with embryos was placed in pre-chilled (-20°C) methanol (Sigma) for 10 mins at -20°C to permeabilize cells, and allowed to air dry for 30 mins at room temperature. Dried samples were then heat shocked with TE buffer, pH 8.0 (Promega) at 70°C for 10 mins and washed with 2X SSC (ThermoFisher Scientific). Samples were hybridized for 6h at 38.5°C in a humidity chamber with XIST Quasar 570 (125 nM; SMF-2038-1; BioSearch Technologies) and ATRX Quasar 670 (125 nM; VSMF-2019-5; BioSearch Technologies) in hybridization buffer comprising of RNase free water, 2X SSC, 10% w/v dextran sulfate (Sigma), 10% formamide (ThermoFisher Scientific), 2 mg/ml E. coli tRNA (Sigma), 2 mM ribonucleoside vanadyl complex (New England Biolabs), and 2 mg/ml bovine serum albumin (Jackson ImmunoResearch). Following hybridization, samples were washed with 20% formamide in 2X SSC for 4x15 mins at 38.5°C. During the last 15 mins wash, Hoescht 33342 (1μg/ml; ThermoFisher Scientific) was added to the wash buffer. Samples were then washed with 2X SSC and mounted with Prolong Diamond antifade (ThermoFisher Scientific) and allowed to dry 24 h in the dark at room temperature before imaging.

DNA FISH

Following RNA FISH, the coverslip with embryos was recovered by 15 mins incubation in PBS at 37°C, and washed in PBS for 5 mins. Samples were then re-fixed with 4% formaldehyde, 0.5% Tergitol (Sigma) and 0.5% Triton X-100 (Sigma) for 10 mins at room temperature, followed by a PBS wash and then incubated for 30 mins at 37°C with 2 mg/ml RNase A (Qiagen). Following a PBS wash, samples were treated with 0.5% Triton X-100 in 0.2 N HCl for 10 mins on ice and denatured in 70% formamide in 2X SSC at 73°C for 7 mins. Following denaturation, samples were immediately placed in chilled 70% Ethanol on ice for 2 mins, followed by 80% and 100% ethanol (2 mins each). Hybridization mix of Vysis CEP Y (DYZ1) Spectrum Orange (05J08-024; AbbottMolecular) and Vysis CEP X (DXZ1) Spectrum Green (05J10-023; AbbottMolecular) in CEP hybridization buffer was denatured in 73°C for 5 mins and applied to samples for 16h at 42°C in a humidity chamber. Following hybridization, samples were washed with 50% formamide in 2X SSC for 2x5 mins, and 2X SSC for 2x5 mins at 46°C. Samples were then stained for Hoescht 33342 (1μg/ml) for 15 mins at room temperature and subsequently washed in 2X SSC. Prolong Diamond antifade mounting media was used and allowed to dry for 24h in the dark at room temperature before imaging.

Imaging
Samples were imaged using Nikon Eclipse Ti-E inverted microscope with 60X 1.4 NA oil-immersion objective, Nikon Intensilight mercury-fiber illuminator, and Andor Zyla 5.5 camera, with 0.3µm z-stack step size.

FISH Image Processing and Analysis
The acquired images where first converted to numpy arrays using the nd2reader python library (Jim, 2015) and then processed with a previously described (Zeisel et al., 2015) custom python script relying on the numpy, scipy.ndimage (Jones et al., 2015) and scikit-image (van der Walt et al., 2014) libraries. Briefly, after background removal using a large kernel gaussian filter, a Laplacian-of-Gaussian was used to enhance the RNA dots. The images were then stitched with Fiji's grid/collection stitching plugin using the nuclei staining as reference (Preibisch et al., 2009). For the *ATRX* quantification, RNA dots were counted blindly with respect to embryonic sex, and to avoid counting RNA specks from potentially overlapping soma only signals within the nuclear perimeter were considered.

Experimental Confirmation of SNPs and Biallelic Expression by Sanger Sequencing
For each SNP confirmation, 2ul of Smart-seq2 cDNA library from a single female E7 cell was used as template, and an amplicon incorporating the SNP of interest was amplified by PCR using KAPA HiFi HotStart ReadyMix (KAPA Biosystems) and the following thermal cycle: 98°C (3 mins), followed by 22 cycles of 98°C (20s), 59°C (15s), 72°C (30s), and a final elongation step 72°C (5 mins). After amplification, the PCR products were purified using Ampure XP beads (Beckman Coulter) at the volume ratio 1:1. The fragment size and yield of product for each cell and amplicon were inspected on an Agilent 2100 Bioanalyzer (Agilent Technologies), and products of confirmed correct size were Sanger sequenced (LIGHTrun, GATC Biotech AG Germany). ApE v2.0.49 (M. Wayne Davis) was used for the sequence alignments. The following primers (5' to 3') were used for the PCRs as well as in the Sanger reactions: *HUWE1* (rs2281481): GCA CAG CAA GGC GAG TAT AC, CCC TGA GAC GAG AGC AAG AA; *MORF4L2* (rs874): ACA CTG GTA GCA ACT TTG AAA TG, AAA GCC CTG TGA GCG TCT AC; *MPP1* (rs1126762): TCT GCA GCT GAT CCA CTG AA, GCG GAA AGT GCG ACT CAT AC; *PRPS1* (rs61752962): AAT TTG TAT GCA GAG CCG GC, AGT GTC AGC CAT GTC ATC CA; *PDHA1* (rs1126565 and rs709610): TCA TTC CTG GGC TGA GAG TG, GCA CTA ATG TAC AAA CTG CAT GC; *TSPAN6* (rs8575): GCT CTT CCA GTG TTT CAG AGG, GTG GGC CTA TTC CTC TCT ACC; *TSPAN6* (rs1802288): GAC ACC ACA ACA ATG CAA CG, CTA CCT GCC GAG CTT CTG; *XIST* (rs1620574): TAG GGC ATG TAG TTC CGA GC, AAA CTG CCA CCC ATA TAT AAG CT.

Single-Cell RNA-Seq Data Pre-Processing and Quality Control
Reads were mapped to the human genome (hg19) using STAR with default settings (Dobin et al., 2013) and only uniquely mapped reads were kept. Gene expression levels (RefSeq annotations) were estimated in terms of reads per kilobase exon model and per million mapped reads (RPKM) using rpkmforgenes (Ramsköld et al., 2009). Read counts from regions where different RefSeq genes overlapped were excluded. Genes were filtered, keeping 15,633 out of 26,178 genes that were expressed in at least 5 out of 1,919 sequenced cells (RPKM $\geq$ 10) and for which cells with expression came from at least two different embryos. Cells were quality-filtered based on four criteria, leaving 1,529 cells post-filtering out of 1,919 sequenced cells. First, Spearman correlations, using the RPKM expression levels of all genes, for every possible pair of cells were calculated and a histogram of the maximum correlation obtained for each cell, corresponding to the most similar cell, was used to identify 305 outlier cells with a maximum pair-wise correlations below 0.63 (Figure 1A). Second, a histogram of the number of expressed genes per cell was used to identify 330 outlier cells with less than 5000 expressed genes (Figure 1B). Third, a histogram of the total transcriptional expression output from the sex chromosomes (RPKM sum) was used to identify 33 cells with indeterminable sex, or a called sex that was inconsistent with other cells of that embryo (see Figure S1 and section "Calling Embryonic Sex", below). Fourth, 13 outlier cells were identified using PCA and t-SNE dimensionality reduction.

Gene Variability and Temporal Separation of Cells
A gene-variability statistic was calculated that adjusted for the mean-variance relationship present in single-cell RNA-seq data (Figure S2A). This was done by assuming that the expression distribution of a gene follow a negative binomial for which the variance depends on the mean, $v = m + m^2/r$, where r is the overdispersion, implying that $cv^2 = v/m^2 = 1/m + 1/r$. To estimate the technical variability we fitted such a model to our ERCC spike-in read counts and a gene-variability statistic was then obtained by adjusting for the technical variability present when conditioning on the mean expression level (Brennecke et al., 2013). To stabilize the estimate we performed winsorization of the expression distribution of each gene, setting the most extreme value to the expression of the second most extreme cell. To tune the number of variable genes used, we plotted the gene-variability statistic versus gene rank (Figure S2B) and performed grid-searches, including 100, 250, 500 and 1000 of the most variable genes, and visually assessed clusters obtained. Temporal separation of cells was

obtained by applying dimensionality reducing techniques, including principal component analysis (PCA), student-t stochastic neighbor embedding (t-SNE; van der Maaten and Hinton, 2008) and diffusion maps (Haghverdi et al., 2015), to all cells using the most variable genes (Figure S2C-D). Cells were assigned a pseudo-time by fitting a principal curve (Hastier and Stuetzle, 1989) to all cells within the subspace spanned by the first two t-SNE dimensions, excluding ICM cells, as to not let lineage segregation affect the temporal principal curve fit (Figure 1F). Subsequently, cells were orthogonally projected onto the fitted principal curve and its unit-speed arc-length parameterization was used as pseudo-time.

Lineage Segregation of Cells

Cells were stratified by embryonic day that they were picked and within each such stratum a PCA was performed. Cells were colored by their expression of previously known lineage markers using a weighted mean, where expression levels of marker genes of one lineage were assigned a weight of 1 and genes of the other lineage a weight of -1 (Figure 2B, 2D and S3). Principal components of interest were identified by both observing a separation of the cells' marker coloring as well as that genes identified to be relevant for lineage separation were found to have high PCA loadings. We adjusted for embryo-wise batch effects in cases where cells were primarily clustering by embryo using COMBAT (Johnson et al., 2007). With respect to E5 cells we observed a subset of cells that were placed prior to a sharp increase in spread in a t-SNE plot of all cells (Figure 2A), and we used a pseudo-time cutoff of ≤ 12.5 (mean pseudo-time per embryo) to indicate such early E5 embryos. Furthermore, these cells were in the middle with respect to PC1, which we identified as the E5 TE-ICM principal component; therefore they were assigned as pre-lineage (Figure 2B). Likewise, a second subset of E5 cells were also assigned as pre-lineage as they were in the middle of the ICM-TE principal component (PC1) and they also formed a middle cluster that exhibited co-expression of ICM and TE genes when performing hierarchical clustering using E5 ICM and TE genes obtained from differential expression analysis (Figure 2C), as described in the next section.

Lineage Differential Expression Analysis

Within each of the three stages, E5-E7, every pair-wise combination of lineage groups, among the three groups of cells corresponding to lineage, EPI, PE and TE, was subjected to single cell differential expression analysis using SCDE and adjusting for sex by supplying it as a co-variate (Kharchenko et al., 2014) (Table S1). Two-sided p-values were calculated from the Benjamini-Hochberg multiple testing corrected Z-score (cZ) using the normal distribution as null hypothesis, and a significance level of 0.05 was used to deem genes as significantly differentially expressed. Lineage-specific p-values were obtained by combining the SCDE cZ-scores for the two corresponding pair-wise comparisons using Stouffer's method. For example, EPI-specific p-values for each gene were derived by combining the two cZ-scores from the EPI vs. PE and EPI vs. TE comparisons. To derive p-values reflecting the tendency for a gene to maintain its lineage-specificity we combined the three lineage-specific p-values from each of the stages, E5-E7, using Stouffer's method (Table S1 and S2). Agglomerative hierarchical clustering using differentially expressed genes as input (Figure 2C, 2E, 4E and S3) was conducted on log10-transformed RPKM expression-values, adding a pseudo-count of 1e-10, and employing Pearson correlation as distance measure between gene-pairs and between cell-pairs and using complete linkage as distance measure between clusters. Genes and cells with constant variance were removed prior to hierarchical clustering. Gene Ontology (GO) gene set enrichment analysis with the top 100 maintained lineage-specific differentially expressed genes from each of the three lineages as input was done using a hypergeometric test and Benjamini-Hochberg multiple testing adjustment (Figure 3C and Table S3). GO terms were retrieved from a local mirror of the GO database.

Lineage Sub-population Analysis

To investigate if there were any subpopulations within the lineages we stratified the cells by embryonic day and lineage, resulting in 9 strata ({E5-E7} x {EPI, PE, TE}), and calculated the most variable genes within each such stratum, accounting for the mean-variance relationship as described above. For each stratum we used the top 250 most variable genes and found that the strongest remaining factor was embryo-to-embryo differences, since cells clearly tended to cluster by embryo, both when using agglomerative hierarchical and when looking at the first two principal components of a PCA plot. We therefore adjusted for embryo effects within each stratum, by supplying embryo as a batch factor to the program Combat (R package "sva") and subsequently retrieved the most variable genes of the embryo-adjusted data within each stratum. We used the top 250 most variable genes, and performed PAM clustering in the PCA dimensionality reduced space (Figure 3D). Once cells had been classified we conducted differential expression analysis between the two groups, separately in E6 and E7, using SCDE, and p-values from E6 and E7 were combined using Stouffer's method (Table S4). Gene Ontology gene set enrichment analysis was subsequently done on the intersection of genes being significantly upregulated in both E6 and E7 in the class denoted as "polar" using genes expressed in all E6 and E7 TE cells as background (7458 genes, mean RPKM ≥ 5 across all cells and RPKM ≥ 5 in $\geq 10\%$ of E6 and E7 TE cells).

Developmental Progression Analysis

To assess temporal differences we conducted differential gene expression analysis between each embryonic day, and between time-points within the same lineage using SCDE (Figure S4A and Table S5). To obtain a view of the complete dataset with respect to lineage segregation and developmental time we applied a diffusion map to all cells using E5 lineage-specific genes derived from the differential expression analysis between lineages described above (Figure 4A and Movie S1). As an alternative approach to obtain a simultaneous view of the lineage segregation and developmental time we plotted the degree of lineage-specificity of each cell versus its embryonic pseudo-time (Figure 4B and 4C). To determine the degree of lineage-specificity of cells we projected all cells to the two diffusion map components where a clear segregation with respect to lineage was observed. Subsequently, we trained a support vector machine (SVM) to find a decision surface that optimally separated the lineages within the subspace of these two lineage-related diffusion map components. The lineage-specificity of each cell was then calculated as a cell's orthogonal distance to the SVM lineage decision surface. Embryonic pseudo-time was calculated as the mean of the cellular pseudo-times of the cells belonging to an embryo. Classification of E5 cells into temporal sub-groups was done using top 300 maintained lineage-specific genes (100 genes from each lineage) and by identifying hierarchical sub-clusters corresponding to specific expression patterns (Figure 4E). Genes were grouped by hierarchical clustering using Pearson's correlation as distance between genes (Figure 4D and 4E). To assess expression variability of genes within embryos and how that variability changed over time (Figure S4B), we first calculated a variability score adjusted for the mean-variance dependency present in single-cell RNA-seq data by calculating the ratio between the squared coefficient of variation (CV^2) for a gene, with respect to the expression distribution within an embryo, and the predicted technical CV^2 obtained from the ERCC spike-in expression levels, as described above. As expression level input we used read counts normalized with respect to the size-factor of each expression library (Love et al., 2014), and for which winsorization of the most extreme expression levels to that of the second most extreme cell, among all 1,529 cells, was done. To avoid a dependency on the number of cells per embryo, we performed quantile normalization between the embryos with respect to each embryo's distribution of such obtained gene-wise variability ratios. To approximately order genes by the time that their expression variability reached a certain level, we performed local linear regression using a 1st degree polynomial and using number of nearest neighbors (NN) as smoothing parameter, since the time-sampling of the embryos was not evenly spaced (R package locfit). Optimal NN was determined by a grid-search tuning of the NN, in the range 0.5 to 1, in steps of 0.01, choosing the model with the smallest Akaike Information Criterion. We timed all maintained lineage genes by the first time-point that a gene's local regression curve reached a particular variability score threshold. The threshold was chosen as 0.83, corresponding to the 90th percentile with respect to the variability score among all RefSeq genes. To assess gene-gene correlation patterns (Figure S4C-D and Table S6) we calculated Pearson's correlation coefficients for RPKM expression levels between all possible pairs of maintained lineage genes. RPKMs were winsorized to stabilize against the most extreme value, censored to 1 RPKM to avoid possible random correlations, and log10-transformed.

Inference of Embryonic Sex

To determine the sex of each cell and embryo, we used the expression of Y-linked genes as indicator of sex. Cells with a chromosome-Y (chrY) RPKM sum ($\Sigma RPKM_{chrY}$) >100 were classified as male and cells with $\Sigma RPKM_{chrY}$ <50 were classified as female. Cells with 50< $\Sigma RPKM_{chrY}$ <100 were excluded form the analyses. This segregated the embryos into two distinct groups (Figure S1A), with a mean $\Sigma RPKM_{chrY}$ of 605 for male-classified cells and 1.0 for female-classified cells. For E3 cells we used a criterion, modified to be somewhat less strict, $\Sigma RPKM_{chrY}$ >100 in at least 50% of the cells of an embryo, to classify the whole embryo as male. The reasoning behind this special criterion in E3 was that the activation chrY genes was apparently incomplete in E3 (mean $\Sigma RPKM_{chrY}$ = 181, standard deviation 97 for male E3 cells and 636, standard deviation 182 for male E4-E7 cells) — in line with the notion of incomplete ZGA at E3. Cells that could not be sex-determined, or cells with a classified sex that was in conflict with other cells in an embryo, were excluded from further analysis (33 cells, 2.2%). For the X-chromosome analyses, we also excluded one female embryo (E5.early.31) that showed signs of an X0 karyotype, *i.e.* Turner syndome (Figure S1E and Figure S1G). For the analyses of *XIST*, *XACT*, and allelic expression, we also excluded a single male embryo (E6.15), as biallelic expression of many X-linked genes was detected in all cells of this particular male-classified embryo (something that we did not observe in any other male embryo).

Sex Differential Expression Analysis

To contrast cells of male and female sex, we performed differential expression analysis using SCDE within each stage and lineage (Table S7). P-values were calculated as described above and a significance level cutoff of 0.05 was used to deem a gene significantly differentially expressed (Figure S4E). To assess the sex differences over time we also calculated the number of genes with an absolute fold-change ≥ 2 within each embryonic day (E4-

E7) (Figure S4F). To avoid any possible dependency on the number of cells per stage we bootstrap resampled the number of cells down to 100 cells within each sex and stage. To investigate chromosome X-Y paralogous gene-pairs we selected such genes (Navarro-Costa, 2012) among significantly differentially expressed genes (Figure S4H) and calculated the correlation between mean male stage-wise expression levels using censored (RPKM ≥ 1) and log10-tranformed RPKM expression values (Figure S4I).

Zygotic Genome Activation and Maternal RNA Clearance

Total chrY expression per cell, shown in Figure 1C, was calculated as: $\Sigma(RPKM_{gene\ i} / \mu_{(1/2)\ gene\ i,\ Male\ E4\text{-}E7})_{ubiquitous}$ in which $RPKM_{gene\ i}$ denotes the expression level (RPKM) of a chrY gene "*i*" in a cell, $\mu_{(1/2)\ gene\ i,\ Male\ E4\text{-}E7}$ denotes the median RPKM for gene "*i*" over all male E4-E7 cells. The sum, $\Sigma(x)_{ubiquitous,}$ was calculated over broadly expressed chrY genes (*DDX3Y*, *EIF1AY*, *KDM5D*, *PRKY*, *RPS4Y1*, *UTY* and *ZFY*), and the inclusion of all chrY genes gave similar results in this analysis. The fraction of chrX SNVs with biallelic calls (Fbi_{chrX}), used as indicator for the presence of maternal RNA in male (XY) embryos was determined as described in the section "Analyses of Allelic Expression" below. Cells with at least 25 informative SNPs on chrX were used in this analysis.

X-chromosome Expression

The distributions of Spearman correlations shown in Figure 5A were calculated using genes with mean RPKM (μ_{RPKM})>5 within each sex using cells from E4 to E7. The gene-wise female-to-male fold-changes shown in Figure 5B-E were calculated as the ratio $\mu_{RPKM,\ female\ cells} / \mu_{RPKM,\ male\ cells}$, including genes with $\mu_{RPKM,\ female\ \&\ male}$>5 over the cells of the given embryonic day and lineage. The moving average of female to male relative expression shown in Figure 5G was calculated using a sliding window of 25 genes, and the ratio $\mu_{(1/2)RPKM,\ female\ cells} / \mu_{(1/2)RPKM,\ male\ cells}$, stratified on stage, in which $\mu_{(1/2)RPKM}$ denotes median RPKM. Other window sizes gave similar results. Genes with $\mu_{(1/2)RPKM}$ >5 in each of the sexes were included in this analysis. *XACT* RPKM (Figure S5A) was calculated using reads aligning to the region chrX:112,983,323-113,235,148, at which we observed a distinct peak of mapped reads above the background levels (Figure S5B). The RPKM sums for chrX shown in Figure 5F were calculated per cell using genes with 5<μ_{RPKM}<200 calculated over all cells and embryonic days (the upper threshold was applied to prevent the weight of a few very high-expressed genes to dominate the estimate). The RPKM sums for human pancreatic alpha cells (Smart-seq2 single-cell RNA-seq data from Athanasia Palasantza and Dr Åsa Segerstolpe (Rickard Sandberg's lab), kindly shared before official data release) were calculated in the same way, but using genes with 5<μ_{RPKM}<200 across pancreatic cells rather than embryonic cells.

Analyses of Allelic Expression

We used SAMtools mpileup (v. 1.2) to retrieve allelic read counts for SNVs available in dbSNP (build 142). Somatic SNVs were excluded (dbSNP flag SAO) and only validated SNVs were included (dbSNP flag VLD; 2+ minor allele count based on frequency or genotype data). Intergenic SNVs were excluded using Annovar (Wang et al., 2010) retaining SNVs within RefSeq genes. dbSNP genomic coordinates were liftover from hg38 to hg19 using the UCSC liftOver command line utility. SNVs identified within *XIST* (Figure 6B) were located in a region where *XIST* and *TSIX* overlap. To rule out the possibility that *TSIX* might have caused a false biallelic detection of *XIST*, we estimated *TSIX*'s contribution to this signal using corresponding *TSIX*- and *XIST*-specific regions (Figure S6C-E). This analysis showed that *TSIX* was unlikely to cause the detected biallelic expression as the number of aligned reads to *TSIX*-specific regions were not above background levels (e.g. *XIST* expression was 431-fold higher than *TSIX* expression in female E7 cells; Figure S6D). The lack of reads mapping to *TSIX*-specific regions in exon-intron overlapping segments was also apparent when visualizing the aligned reads using the IGV Genome browser (Broad Institute) (Figure S6C). For calling allelic expression (as either undetected, biallelic, or monoallelic with respect to the reference or alternative allele), we required at least three reads to call an SNV locus expressed, and an allele-specific expression bias of at least 10-fold to call an SNV locus monoallelic. The reasoning behind using the 10-fold criterion, rather than a 50-fold difference used in an earlier study of allelic expression (Deng et al., 2014a), was to attain higher sensitivity to detect allele-biased states. With either criterion we observed that the rate of biallelic chrX expression in human female E4-E7 cells was similar to that of autosomes. For comparability, the plots of allelic expression in mouse, shown in Figure S6E-F, were based on the same criteria as for the human allelic analyses. The biallelic ratios shown in Figure 6C and Figure S6F were calculated as $Fbi_{chrX} / Fbi_{autosomes}$, in which *Fbi* denotes the fraction of SNVs with biallelic calls (biallelic calls / (biallelic calls + monoallelic calls)) in each cell. Cells with at least 25 SNP calls (bi- or mono-allelic) on chrX were considered in Figure 6C as to only include cells with reliable estimates of the fraction of biallelic calls, and stricter or looser criteria for cell inclusion (e.g. minimum 40 or 20 allelic SNP calls) provided similar results. The same calculations were performed for chr1-3 (Figure 6C).

Supplemental References

Dobin, A., Davis, C.A., Schlesinger, F., Drenkow, J., Zaleski, C., Jha, S., Batut, P., Chaisson, M., and Gingeras, T.R. (2013). STAR: ultrafast universal RNA-seq aligner. Bioinformatics *29*, 15–21.

Jim, R. (2015). nd2reader 2.0.0.

Johnson, W.E., Li, C., and Rabinovic, A. (2007). Adjusting batch effects in microarray expression data using empirical Bayes methods. Biostatistics *8*, 118–127.

Jones E, Oliphant E, Peterson P, et al. SciPy: Open Source Scientific Tools for Python, 2001-, http://www.scipy.org/ [Online; accessed 2015-12-16].

Navarro-Costa, P. (2012). Sex, rebellion and decadence: the scandalous evolutionary history of the human Y chromosome. Biochim. Biophys. Acta *1822*, 1851–1863.

Preibisch, S., Saalfeld, S., and Tomancak, P. (2009). Globally optimal stitching of tiled 3D microscopic image acquisitions. Bioinformatics *25*, 1463–1465.

Van der Walt, S., Schönberger, J.L., Nunez-Iglesias, J., Boulogne, F., Warner, J.D., Yager, N., Gouillart, E., and Yu, T. (2014). scikit-image: image processing in Python. PeerJ *2*, e453.

Zeisel, A., Manchado, A.B.M., Codeluppi, S., Lonnerberg, P., La Manno, G., Jureus, A., Marques, S., Munguba, H., He, L., Betsholtz, C., et al. (2015). Cell types in the mouse cortex and hippocampus revealed by single-cell RNA-seq. Science (80-.). *347*, 1138–1142.
