# Supplementary figures and images for: Single-Cell RNA-Seq Reveals Lineage and X Chromosome Dynamics in Human Preimplantation Embryos
Source: Cell. 2016 May 5;165(4):1012–26. doi: 10.1016/j.cell.2016.03.023 (PMC4868821; doi:10.1016/j.cell.2016.03.023)

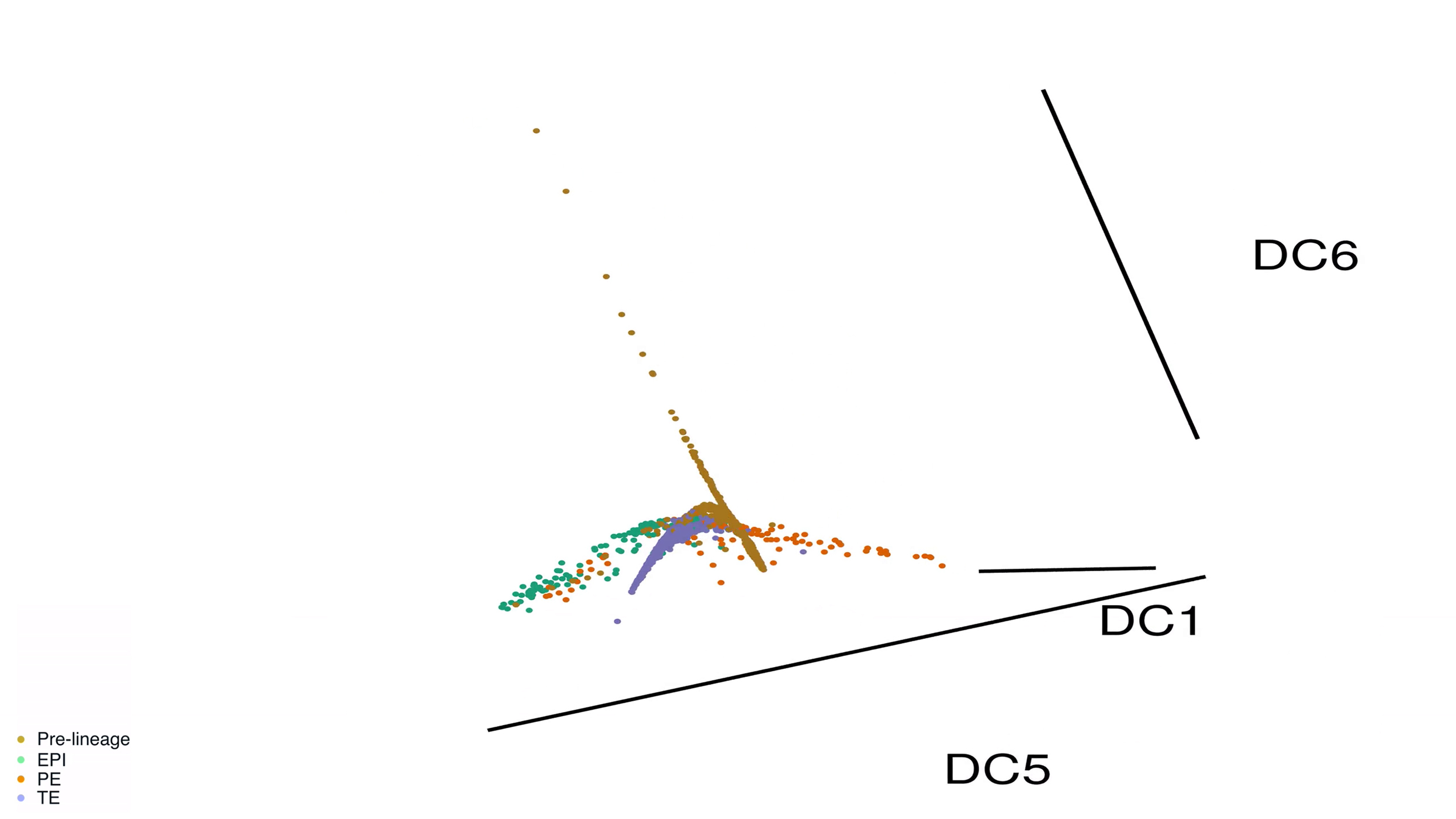

Supplement: Movie S1. Preimplantation Temporal Progression and Lineage Segregation, Related to Figure 4 — Three-dimensional diffusion map representation of all cells using 94 lineage-specific genes at E5 as input. [file mmc9.jpg]
